# Autocatalytic bifunctional supramolecular hydrogels for osteoporotic bone repair

**DOI:** 10.1093/nsr/nwae209

**Published:** 2024-06-20

**Authors:** Zhihui Han, Xiang Gao, Yuanjie Wang, Cheng Huang, Hao Song, Shuning Cheng, Xiaoyuan Yang, Xiaoliang Cui, Jie Wu, Kailu Wei, Liang Cheng

**Affiliations:** Institute of Functional Nano & Soft Materials (FUNSOM), Collaborative Innovation Center of Suzhou Nano Science and Technology, Soochow University, Suzhou 215123, China; Department of Orthopedics, The Second Affiliated Hospital of Soochow University, Suzhou 215004, China; Institute of Functional Nano & Soft Materials (FUNSOM), Collaborative Innovation Center of Suzhou Nano Science and Technology, Soochow University, Suzhou 215123, China; Department of Orthopedics, The Second Affiliated Hospital of Soochow University, Suzhou 215004, China; Department of Orthopedics, The Second Affiliated Hospital of Soochow University, Suzhou 215004, China; Institute of Functional Nano & Soft Materials (FUNSOM), Collaborative Innovation Center of Suzhou Nano Science and Technology, Soochow University, Suzhou 215123, China; Institute of Functional Nano & Soft Materials (FUNSOM), Collaborative Innovation Center of Suzhou Nano Science and Technology, Soochow University, Suzhou 215123, China; Institute of Functional Nano & Soft Materials (FUNSOM), Collaborative Innovation Center of Suzhou Nano Science and Technology, Soochow University, Suzhou 215123, China; Institute of Functional Nano & Soft Materials (FUNSOM), Collaborative Innovation Center of Suzhou Nano Science and Technology, Soochow University, Suzhou 215123, China; Institute of Functional Nano & Soft Materials (FUNSOM), Collaborative Innovation Center of Suzhou Nano Science and Technology, Soochow University, Suzhou 215123, China; Institute of Functional Nano & Soft Materials (FUNSOM), Collaborative Innovation Center of Suzhou Nano Science and Technology, Soochow University, Suzhou 215123, China; Macao Institute of Materials Science and Engineering, Macau University of Science and Technology, Taipa 999078, China

**Keywords:** autocatalysis, supramolecular hydrogel, anti-inflammation, osteoporosis, bone defect

## Abstract

Conventional bone scaffolds, which are mainly ascribed to highly active osteoclasts and an inflammatory microenvironment with high levels of reactive oxygen species and pro-inflammatory factors, barely satisfy osteoporotic defect repair. Herein, multifunctional self-assembled supramolecular fiber hydrogels (Ce–Aln gel) consisting of alendronate (Aln) and cerium (Ce) ions were constructed for osteoporotic bone defect repair. Based on the reversible interaction and polyvalent cerium ions, the Ce–Aln gel, which was mainly composed of ionic coordination and hydrogen bonds, displayed good injectability and autocatalytic amplification of the antioxidant effect. *In vitro* studies showed that the Ce–Aln gel effectively maintained the biological function of osteoblasts by regulating redox homeostasis and improved the inflammatory microenvironment to enhance the inhibitory effect on osteoclasts. Ribonucleic acid (RNA) sequencing further revealed significant downregulation of various metabolic pathways, including apoptosis signaling, hypoxia metabolism and tumor necrosis factor-alpha (TNF-α) signaling via the nuclear factor kappa-B pathway after treatment with the Ce–Aln gel. *In vivo* experiments showed that the clinical drug-based Ce–Aln gel effectively promoted the tissue repair of osteoporotic bone defects by improving inflammation and inhibiting osteoclast formation at the defect. Notably, *in vivo* systemic osteoporosis was significantly ameliorated, highlighting the strong potential of clinical translation for precise therapy of bone defects.

## Teaser

Multifunctional self-assembled supramolecular fiber hydrogels consisting of clinical drug alendronate and cerium ions with the autocatalytic antioxidant amplification exhibit excellent therapy on osteoporotic bone defect and systemic bone loss.

## INTRODUCTION

Osteoporosis—a progressive skeletal disease—has become a global health issue [1,2] and osteoporosis patients, especially postmenopausal women, are more susceptible to local fractures in daily life due to systemic bone loss and microstructural destruction [3–5]. However, a more difficult recovery situation is faced by fracture repair under osteoporosis in that the overactive osteoclasts have broken the original balance of the bone formation and resorption metabolism, which means that traditional filling implants lacking functionality, such as polymethyl methacrylate (PMMA), cannot satisfy the effective treatment for the injury [6,7]. Therefore, it is necessary to confer the ability to fill implants to inhibit the activity of osteoclasts for the treatment of osteoporotic bone defects. Alendronate (Aln)—a Food and Drug Administration-approved drug—has been verified to affect the activity of osteoclasts due to its unique ‘–P–C–P–’ structure [8]. Unfortunately, the intravenous/oral mode of administration resulted in a limited enrichment of Aln at the local defect site, and the increasing dose may lead to adverse drug reactions such as hypocalcemia and nephrotoxicity [9,10]. Hence, it is essential and feasible to establish a controlled and safe local delivery system to improve drug efficacy and reduce side effects.

On the other hand, it has been reported that osteoporosis is accompanied by chronic inflammation [11,12]. The persistent free radicals at the bone defect directly destroy osteoblast homeostasis and interfere with physiological functions [13,14]. Meanwhile, the high levels of pro-inflammatory cytokines released from immune cells, such as tumor necrosis factor-α (TNF-α) and interleukin-6 (IL-6), induce the expression of the receptor activator of nuclear kappa-B ligand (RANKL) by activating the nuclear factor kappa-B (NF-κB) pathway in macrophages, which then promotes the formation and differentiation of osteoclasts [15]. Hence, the regulation of the inflammatory environment cannot be ignored in the balance of bone metabolism in osteoporosis, which means that filling implants need to take antioxidant and anti-inflammatory capabilities into consideration. Therefore, newer and higher demands are required for local drug-loaded systems. Unfortunately, the satisfaction of these requirements usually means more complex design and construction processes, which attract technical challenges, high manufacturing costs and inefficiency issues resulting from the complex synthesis process, as well as poor stability and *in vivo* potential toxicity issues that hinder the feasibility of clinical translation [16–18]. Therefore, it is highly desirable to develop a convenient and biocompatible hydrogel with multiple functions to repair osteoporotic bone defects.

**Scheme 1.**
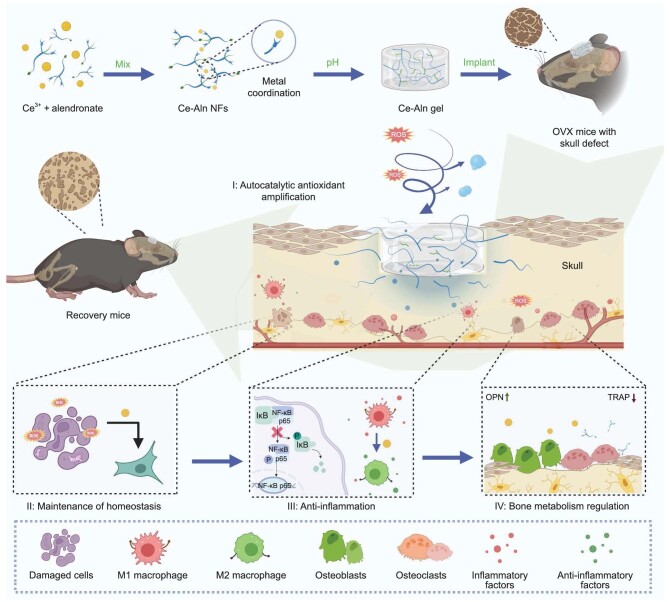
Schematic illustration of osteogenic enhancement and osteoclastic inhibition by the Ce–Aln gel. The Ce–Aln gel was autocatalysed to enhance the antioxidant capacity in response to ROS in the bone defect environment, cleared ROS to produce oxygen and maintained redox homeostasis in osteoblasts to avoid apoptosis. It inhibited the production of pro-inflammatory macrophages and pro-inflammatory factors to improve the inflammatory microenvironment and promote bone formation, further enhancing the ability of alendronate to inhibit osteoclasts and reverse the *in vivo* imbalance of bone metabolism (created with BioRender.com).

Hence, considering the unique chemical molecular structure of Aln and its high coordination affinity for metal ions [19,20], we constructed a bifunctional supramolecular fibrous hydrogel for the treatment of osteoporotic bone defects (Scheme 1). Cerium ion was selected for coordination with Aln because of its good antioxidant properties and biosafety to effectively combat the inflammatory microenvironment. The obtained fibrous hydrogel (Ce–Aln gel) was self-assembled into the 3D network under a pH response and derived from the reversible interaction of the clinical drug Aln and Ce ions. Notably, the Ce–Aln gel with good self-healing and injectability displayed autocatalytic amplification of the antioxidant effect in response to reactive oxygen species (ROS). *In vitro* experiments showed that the Ce–Aln gel scavenged excessive ROS to reduce damage to cells and maintain redox homeostasis and normal physiological function. Meanwhile, the Ce–Aln gel regulated the polarization phenotype of macrophages by inhibiting the activated NF-κB pathway, promoting the release of anti-inflammatory factors and reducing pro-inflammatory factors to improve the inflammatory microenvironment, thereby further relieving the imbalance of bone metabolism by enhancing osteogenic differentiation and osteoclast inhibition. Ribonucleic acid (RNA) sequencing further demonstrated that apoptosis signaling, hypoxia metabolism and TNF-α signaling via NF-κB pathway were significantly downregulated with the Ce–Aln gel treatment. Furthermore, the Ce–Aln gel had an excellent bone repair effect after local implantation in ovariectomized (OVX) mice with skull defects. Notably, the systemic osteoporosis was also significantly improved. Thus, our work offers a novel hydrogel with high translational promise for osteoporotic bone defect repair.

## RESULTS AND DISCUSSION

### Synthesis and characterization of Ce–Aln fibers

**Figure 1.**
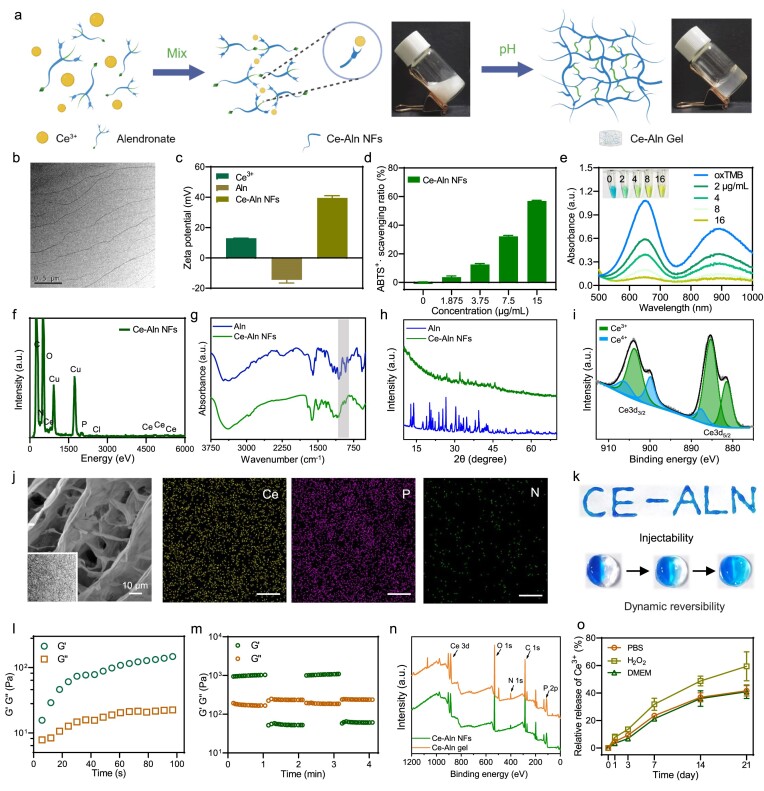
Synthesis and characterization of the Ce–Aln NFs and Ce–Aln gel. (a) Schematic illustration of Ce–Aln NFs and *in vitro* pH-responsive self-assembled Ce–Aln gel (created with BioRender.com). (b) Transmission electron microscopy (TEM) image of Ce–Aln NFs. (c) Zeta potentials of Ce^3+^, alendronate (Aln) and Ce–Aln NFs. (d) ABTS^+^· scavenging ability of Ce–Aln NFs at various concentrations (*n* = 3). (e) Ultraviolet-visible-near infrared (UV-vis-NIR) spectra of Ce–Aln NFs probed with the ox-TMB probe at different concentrations (*n* = 3). (f) Energy dispersive X-ray spectrometry (EDS) spectrum of Ce–Aln NFs, (g) Fourier transform infrared (FT–IR) spectra and (h) X-ray diffraction (XRD) patterns of Aln and Ce–Aln NFs. (i) X-ray photoelectron spectroscopy (XPS) spectrum of Ce–Aln NFs. (j) Scanning electron microscopy (SEM) image of the Ce–Aln gel. The insert map is a TEM image and EDS of the Ce–Aln gel. (k) Photographs showing the injectability and dynamic reversibility of the Ce–Aln gel. (l) The time-dependent rheological curve of the Ce-Aln gel (the crossing point was the ‘gel-forming point’). (m) Self-healing test of the Ce–Aln gel by using a rotational rheometer. The strains were 2% and 20%, respectively. (n) XPS spectra of Ce–Aln NFs and Ce–Aln gel. (o) Relative cerium ion release from the Ce–Aln gel in different media (PBS, H_2_O_2_, DMEM) (*n* = 3).

Ce–Aln coordination nanofibers (NFs) were prepared through a simple one-step self-assembly process (Fig. 1a). By the interaction of the phosphate and amino groups in alendronate (Aln) with Ce ions, Ce–Aln fibers were formed through metal coordination bonds. Transmission electron microscopy (TEM) images showed that short and thick nanoribbons with a diameter of 72 ± 1.6 nm were formed at a Ce^3+^ : Aln ratio of 0.5 : 1. Upon increasing the concentration of Ce^3+^, a nanowire morphology with a diameter of 50 ± 1.6 nm was formed when the ratio of Ce^3+^:Aln increased to 1:1, and the long and slender NFs with diameters of 15 ± 1.7 nm were synthesized when the ratio of Ce^3+^:Aln increased to 2:1. Therefore, a higher concentration of Ce^3+^ was better for the longer fiber formation (Fig. S1 in the online supplementary file and Fig. 1b). All the obtained fibers were positively charged for good cell adhesion and membrane penetration, due to the negative charge of the cell membrane (Fig. S2 and Fig. 1c) [21]. Subsequently, through the use of typical 2,2'-azinobis-(3-ethylbenzthiazoline-6-sulphonate, ABTS) and oxidized 3,3′,5,5′-tetramethylbenzidine (ox-TMB) probes, all the synthesized Ce–Aln coordination fibers were found to possess a strong concentration-dependent ROS-scavenging effect, which was attributed to the increase in Ce ions (Fig. 1d and e, and Fig. S3). Therefore, Ce–Aln NFs with a Ce^3+^:Aln ratio of 2:1 were selected as the representative samples for the further characterization. Energy dispersive X-ray spectrometry (EDS) showed that the Ce–Aln NFs consisted of elements of Ce, P, C, N and O (Fig. 1f), demonstrating that Ce ions were coordinated inside the fibers. Compared with those of Aln in the Fourier transform infrared spectroscopy (FT–IR) analysis, the peaks located at 900–1100 cm^−1^ of the Ce–Aln NFs were significantly weaker and blue-shifted, suggesting that the phosphate groups were involved in coordination bonding with the Ce ions (Fig. 1g). In addition, the O–H in-plane bending vibration from 1500 to 1600 cm^−1^ showed a significant blue-shift, demonstrating hydrogen bond formation after coordination polymerization [22]. Similarly, X-ray diffraction (XRD) analysis revealed that the obvious sharp peaks of Aln in the range of 10–45° were weakened or even disappeared after coordination with Ce ions (Fig. 1h), indicating the formation of an amorphous state of Ce–Aln complex [23]. Furthermore, X-ray photoelectron spectroscopy (XPS) analysis showed that Ce^3+^ and Ce^4+^ co-existed in the Ce–Aln NFs (Fig. 1i), providing the chemical basis for the catalytic activities [24,25]. No peak at a binding energy of ∼917.3 eV (associated with Ce^4+^) was found, indicating the absence of CeO_2_ species [26]. Above all, Ce–Aln NFs consisting of mixed states of Ce^3+^ and Ce^4+^ were successfully constructed and the mixed valances of Ce^3+^ and Ce^4+^ were responsible for the high antioxidative activity in the ROS-scavenging process. In addition, the Ce–Aln NFs showed good biocompatibility without any hemolysis (Fig. S4).

### Synthesis and characterization of Ce–Aln gel

The coordination bond formed after metal coordination is easily disturbed by changes in the external temperature or pH [27–30]. Therefore, a hydrogel with a 3D structure was constructed by adjusting the pH of the environment after obtaining Ce–Aln NFs. Among the different ratios of Ce–Aln complexes, the rheological measurements showed that only Ce^3+^:Aln NFs with a ratio of 2:1 maintained a good solid state with viscoelasticity when the angular frequency increased from 0.1 to 100 rad/s (Figs S5 and S6a) and the nanofiber-to-hydrogel transformation immediately occurred within ∼6 s (Fig. 1l). With the extension of time, the continuous increase in the storage modulus G′ and loss modulus G″ showed that the self-assembly process inside the gel persisted as the hardness of the Ce–Aln fiber increased. Moreover, the Ce–Aln NFs solution changed from a milky white flow to a transparent solid after the pH changed (Fig. 1a).

Based on the dynamic reversible property of the metal coordination bonds, the synthesized fiber gel displayed favorable injectability and dynamic reversibility (Fig. 1k). The oscillation strain-scanning curve showed that the structure of the Ce–Aln gel was damaged when the oscillation strain increased to 19% (Fig. S6b). However, the Ce–Aln gel was rebuilt at a strain of ∼2% and the applied strains of 2% and 20% caused the gel to undergo constant destruction and reconstruction (Fig. 1m), suggesting that the Ce–Aln gel possessed self-healing capacity. TEM and scanning electron microscopy (SEM) images displayed an obvious cross-linking morphology of the 3D layered scaffold (Fig. 1j). In addition, the co-existence of Ce, P, C, O and N was also revealed by using XPS analysis and EDS elemental mapping when the pH was adjusted to import Na (Fig. 1n and Fig. S7). Furthermore, the phosphate group between 900 and 1100 cm^−1^ continued to weaken and blue-shift during the self-assembly process of the gel (Fig. S8), indicating that the pH changes further promoted coordination and new hydrogen bond formation, which was derived from the deprotonation of the amino group and the phosphate group in Aln. Moreover, the Ce–Aln gel gradually transformed into a fluid state when the ethylenediaminetetraacetic acid (EDTA) chelator was added (Fig. S9), strongly confirming the key role of coordination in the hydrogel network [23,31]. Meanwhile, the Ce–Aln gel showed a similar degradation and release rate (∼45%) in the phosphate buffered saline (PBS) and Dulbecco's modified Eagle medium (DMEM) solutions within 21 days (Fig. 1o and Fig. S10), providing the basis for long-term tissue-engineering applications. Interestingly, when hydrogen peroxide (H_2_O_2_) was added to the PBS to mimic the ROS microenvironment, the degradation rate of the Ce–Aln gel and the release rate of the Ce in the Ce–Aln gel significantly increased, demonstrating that ROS interacted with the Ce–Aln gel to accelerate degradation and Ce ion release. Moreover, the Ce–Aln gel was degraded by ∼35% after 7 days and ∼74% after 21 days for hypodermic implantation. Meanwhile, hematoxylin-eosin (H&E)-stained skin tissue on Day 7 showed that almost no inflammatory reaction was observed in the skin tissue of the mice after Ce–Aln gel implantation (Fig. S11). Taken together, a pH-responsive Ce–Aln hydrogel with good mechanical properties and degradability was successfully synthesized.

### Autocatalytic antioxidant amplification capacity and underlying mechanism of the Ce–Aln gel

**Figure 2.**
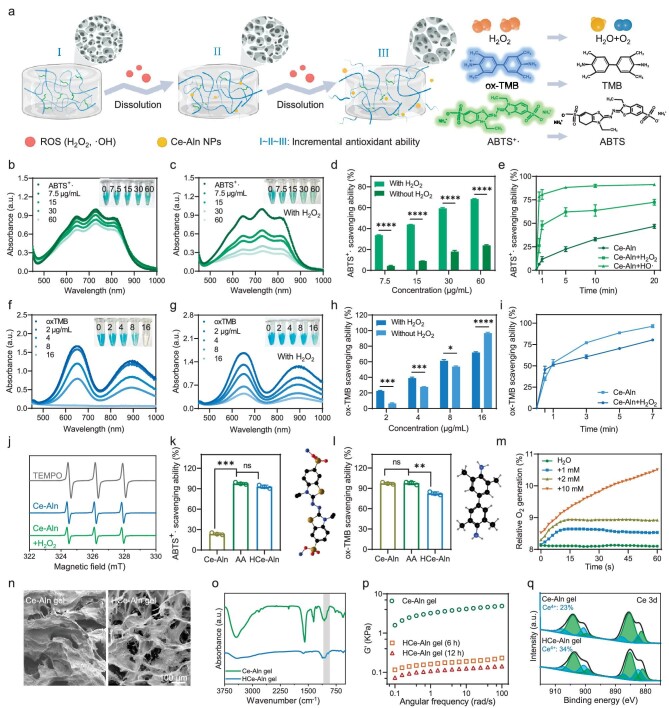
Characterization and mechanism of ROS-induced antioxidant amplification capacity. (a) Schematic illustration of the amplification mechanism of the autocatalytic antioxidation of the Ce–Aln gel (created with BioRender.com). (b) UV-vis–NIR spectra of the Ce–Aln gel and (c) the Ce–Aln gel after treatment with hydrogen peroxide (H_2_O_2_) through ABTS^+^·. The inset map shows the macroscopic color change. (d) Quantification and (e) time-dependent investigation of the ABTS^+^· scavenging capacity of the Ce–Aln gel with/without pretreatment at different concentrations (*n* = 3). (f) UV-vis–NIR spectra of the Ce–Aln gel and (g) the Ce–Aln gel after pretreatment with H_2_O_2_. The inset map shows the macroscopic color change. (h) Quantification and (i) time-dependent investigation of the ox-TMB scavenging capacity of the Ce–Aln gel with/without pretreatment with H_2_O_2_ at different concentrations (*n* = 3). (j) Electron spin resonance (ESR) spectra showing the antioxidant ability of the Ce–Aln gel with/without pretreatment, in which 2,2,6,6-tetramethylpiperidine-1-oxyl (TEMPO) was used as the probe. (k) Quantification of the ABTS^+^· and (l) the ox-TMB scavenging capacities of the Ce–Aln gel, the Ce–Aln gel after treatment with H_2_O_2_ (HCe–Aln gel) and ascorbic acid (AA) at the same concentration. The right panel shows the molecular formula of the probe (*n* = 3). (m) The detection of oxygen production by the Ce–Aln gel with various pretreatments over time (*n* = 3). (n) SEM images and (o) FT–IR spectra of the Ce–Aln gel and HCe–Aln gel. (p) Angular frequency-scan curves of the Ce–Aln gel after various pretreatments (*n* = 3). (q) XPS spectra of the Ce–Aln gel and HCe–Aln gel. *P < 0.05; **P < 0.01; ***P < 0.001; ****P < 0.0001 as determined by using a Student's *t*-test; ns, not significant.

The hypoxic and highly oxidative stress microenvironment of osteoporotic bone defects requires the implanted hydrogels with antioxidant properties. Considering the good antioxidant capacity of Ce–Aln NFs, the ROS-scavenging ability of the Ce–Aln gel was investigated (Fig. 2b and f). The Ce–Aln gel exhibited a better scavenging effect on hydroxyl radicals (·OH), which possessed the most reactivity among free radicals and promoted the valence change of Ce ions in the Ce–Aln gel, resulting in the improvement in the ROS-scavenging effect. To verify this hypothesis, the Ce–Aln gel was pretreated with hydrogen peroxide (H_2_O_2_) or ·OH and then mixed with ABTS^+^· radicals. The free-radical scavenging effect of the Ce–Aln gel was greatly improved (Fig. 2c and d). The scavenging rate of the Ce–Aln gel pretreated with ·OH was ∼7-fold higher than that of the untreated gel after 1 min of the reaction (Fig. 2e). Moreover, a similar treatment occurred when the ox-TMB probe was used for detection. However, the antioxidant effect of the pretreated gel gradually decreased with increasing concentration (Fig. 2g and h) and the ox-TMB scavenging rate of the Ce–Aln gel showed little change after pretreatment (Fig. 2i). This might be because the H_2_O_2_ used for the pretreatment was likewise one of the substrates that generated ·OH during ox-TMB detection. Therefore, electron spin resonance (ESR) was applied to verify the autocatalytic antioxidant effect of the Ce–Aln gel after H_2_O_2_ pretreatment (Fig. 2j). Furthermore, ascorbic acid (AA), a common antioxidant, was used as a control to evaluate and compare its antioxidant capacity. The H_2_O_2_-pretreated Ce–Aln gel (HCe–Aln gel) exhibited a similar antioxidant capacity to that of AA at the same concentration (Fig. 2k), which effectively showed the good autocatalytic antioxidation amplification of the Ce–Aln gel. However, the HCe–Aln gel did not achieve the same effect on the detection of the ox-TMB probe, which may be due to the previously mentioned enhancement of ·OH by H_2_O_2_ (Fig. 2l). At the same time, the Ce–Aln gel effectively catalysed H_2_O_2_ to generate oxygen and the rate of O_2_ generation significantly enhanced with increasing H_2_O_2_ concentrations (Fig. 2m).

Next, the mechanism of ROS-induced autocatalytic antioxidation amplification was explored. SEM images showed that the HCe–Aln gel was exposed at the edges with expanded pores and the porosity of the Ce–Aln gel significantly increased from ∼65% to ∼85% (Fig. 2n and Fig. S12). Moreover, various concentrations of H_2_O_2_ and different kinds of free radicals, such as ABTS^+^· and ·OH, also destroyed the structure of the Ce–Aln gel (Fig. S13). All this evidence indicated that ROS destroyed the Ce–Aln gel structure and enhanced the surface roughness and pore size, which was more conducive to cell adhesion and vessel in-growth [32,33]. TEM images showed that the network structure of the Ce–Aln gel decomposed into NFs and even gradually dissociated into nanodots after incubation with H_2_O_2_ for a long time (Figs S14 and S15). More interestingly, the initially obtained Ce–Aln NFs also displayed better antioxidant effects after pretreatment with H_2_O_2_ (Fig. S16). Furthermore, the characteristic peak of the phosphate group of the HCe–Aln gel at 900–1100 cm^−1^ blue-shifted, indicating the disappearance of the previous bond, which corresponded to the appearance of fibers during the degradation of the HCe–Aln gel (Fig. 2o). A similar result was also revealed by rheological mechanical measurement. The HCe–Aln gel displayed a lower storage modulus G′ after reacting with H_2_O_2_, indicating decreased hardness and a collapsed structure (Fig. 2p). In addition, XPS analysis confirmed the effect of ROS on the valence state of Ce ions in the HCe–Aln gel (Fig. 2q). The ratio of Ce^4+^ to Ce^3+^ significantly decreased, corresponding to the ratio of Ce^3+^ to Ce^4+^ in CeO_2_ with high activity [25]. All this evidence indicated that the autocatalytic amplification of the antioxidant capacity of the Ce–Aln gel was derived from the mechanism by which ROS destroyed the original ionic coordination bonds, resulting in the accelerated degradation of the gel and the release of Ce–Aln NFs with good antioxidant effects. During the reaction with ROS, the increased proportion of tetravalent Ce ions in the gel endowed them with unique antioxidant activity (Fig. 2a). Therefore, the Ce–Aln gels obtained stronger antioxidant effects while scavenging ROS, thus enhancing their ability to optimize the inflammatory immune microenvironment.

### Cytocompatibility, proliferation and maintenance of homeostasis

**Figure 3.**
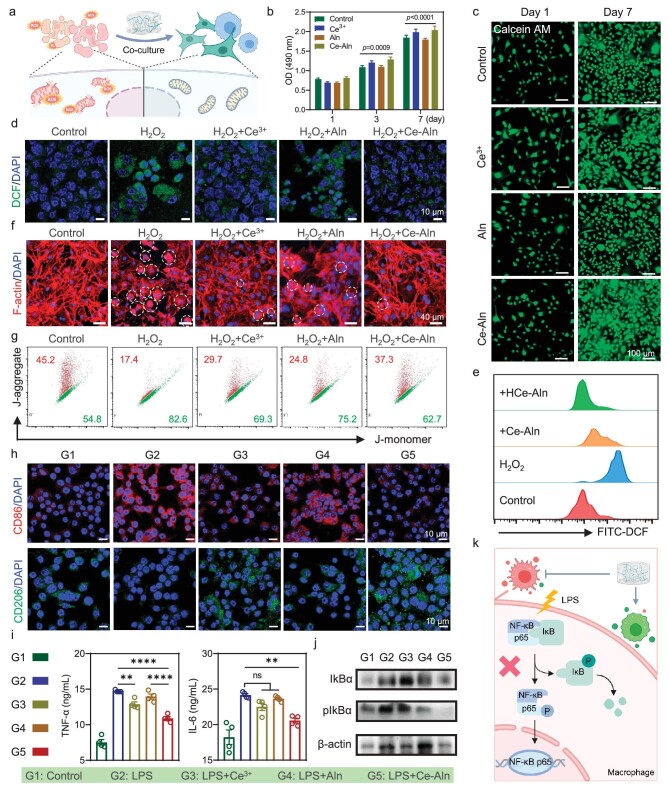
Proliferation, maintenance of homeostasis and macrophage polarization of the Ce–Aln gel. (a) Schematic illustration of changes in cells after co-incubation with the Ce–Aln gel in the presence of ROS (created with BioRender.com). (b) Cell viability of RAW264.7 cells after various treatments at 1, 3 and 7 days (*n* = 6). (c) CLSM observation of MC3T3-E1 cells stained with Calcein AM at 1 and 7 days after various treatments. Scale bar: 100 μm. (d) CLSM images of RAW264.7 cells after various treatments stained with DCFH-DA and 4′,6-diamidino-2-phenylindole (DAPI). Scale bar: 10 μm. (e) Fluorescence activated cell sorting (FACS) results showing intracellular ROS in MC3T3-E1 cells stained with DCFH-DA after various treatments. (f) CLSM images of morphological changes in MC3T3-E1 cells after different treatments stained with phalloidin. Scale bar: 40 μm. (g) FACS results of the mitochondrial membrane potential (MMP) in MC3T3-E1 cells after different treatments. (*n* = 5). (h) CLSM images of RAW264.7 cells after different treatments stained with CD86 (red), CD206 (green) and DAPI (blue). Scale bar: 10 μm. (i) The expression of inflammatory factors TNF-α and IL-6 in macrophages after different treatments by using an enzyme-linked immunosorbent assay (ELISA) kit (*n* = 5). (j) Expression of the NF-κB pathway related proteins in RAW264.7 cells after different treatments, as determined by using Western blotting (WB). (k) Schematic illustration of the modulation of macrophage phenotype by the Ce–Aln gel through inhibition of the NF-κB pathway activated by LPS. The phosphorylation of NF-κB p65 and IκB was inhibited (created with BioRender.com). *P < 0.05; **P < 0.01; ***P < 0.001; ****P < 0.0001, as determined by using Student's *t*-test; ns, not significant.

For clinical bone tissue engineering implantation, the most basic requirement for hydrogels is favorable biocompatibility [34]. Considering the crucial role of the inflammation and bone-remodeling stages in tissue recovery, mouse embryo osteoblast precursor (MC3T3-E1) and mouse leukemic monocyte macrophage (RAW 264.7) cells were co-cultured with the Ce–Aln gel for 24 h, respectively, which exhibited good cytocompatibility according to the standardized 3-(4,5-dimethylthiazol-2-yl)-2,5-diphenyltetrazolium bromide (MTT) assay (Fig. S17). The intrinsic morphology of the MC3T3-E1 cells was maintained and no cell shrinkage occurred after treatment with the Ce–Aln gel (Fig. S18). In addition, the Ce–Aln gel had no influence on the normal rate of proliferation after co-incubation with both RAW 264.7 and MC3T3-E1 cells, even though the Ce–Aln gel had a certain proliferative effect because of Ce ions (Fig. 3b and c, and Fig. S19). Since the acute inflammatory response at the fracture site led to a high oxidative stress, H_2_O_2_ was added as the excess ROS in the microenvironment to verify the antioxidant effect of the Ce–Aln gel. Moreover, a 2′,7′-dichlorodihydrofluorescein diacetate (DCFH-DA) probe was applied to detect intracellular ROS levels. The green fluorescence in RAW264.7 cells was enhanced after incubation with H_2_O_2_ and significantly reduced after Ce–Aln gel treatment due to the strong scavenging effect derived from Ce ions (Fig. 3d). Compared with the Ce–Aln gel, the HCe–Aln gel possessed a better free-radical scavenging ability (Fig. S20). Similar results were observed in MC3T3-E1 cells through confocal laser scanning microscopy (CLSM) and flow cytometry (Fig. S21 and Fig. 3e). Moreover, the catalytic oxygen production capacity of the Ce–Aln gel was evaluated at the cellular level. Using ruthenium tripyridine (Rhb_2_d) as an oxygen probe, the red fluorescence of cells incubated with Ce^3+^ and gel was weaker than that of the control and H_2_O_2_ groups, indicating that oxygen was produced in these two groups (Fig. S22) and the catalytic efficiency of the Ce–Aln gel was significantly greater than that of Ce^3+^.

High oxidative stress often disrupts the original homeostatic level of cells and interferes with the normal physiological function of cells [35]. MC3T3-E1 and RAW264.7 cells treated with H_2_O_2_ achieved a higher survival rate after co-incubation with Ce–Aln NFs, which was attributed to the favorable antioxidant properties of Ce ions (Fig. S23). Furthermore, as fibroblast-like cells, MC3T3-E1 cells normally need to extend pseudopodia for favorable adhesion to the bone surface. Therefore, it is essential to maintain the existence of the pseudopodia. However, oxidative stress led to the shrinkage of the cytoskeletal structure and disappearance of the pseudopodia, which was reversed by the antioxidant capacity of the Ce–Aln gel (Fig. 3f). Moreover, high oxidative stress also causes mitochondrial dysfunction, in which a change in the mitochondrial membrane potential (MMP) is considered to be a signal of the early apoptosis stage. Therefore, JC-1, a staining reagent for MMP, was used to monitor the effect of oxidative stress on the intracellular mitochondrial status. After co-culture with the Ce–Aln gel, the number of MC3T3-E1 cells labeled with red fluorescence significantly increased (Fig. 3g), while the number of cells treated with H_2_O_2_ showed more green fluorescence, indicating that the Ce–Aln gel reduced the mitochondrial damage caused by free radicals and prevented programmed cell death (Fig. S24). Moreover, Western blotting (WB) analysis revealed that H_2_O_2_ upregulated the expression of the apoptosis-related protein Bcl2-associated X (Bax), while the Ce–Aln gel effectively reduced the secretion of Bax (Fig. S25). All these results confirmed that the Ce–Aln gel, with excellent biocompatibility and antioxidant properties, effectively reversed the loss of function and mitochondrial damage caused by oxidative stress in osteoblasts to maintain cellular homeostasis (Fig. 3a).

### Ce–Aln gel regulates the polarization phenotype of macrophages and cytokine release

During the formation of new bone, the dysregulated immune response often creates a conflict for repair, especially for macrophages [36,37]. Lipopolysaccharide (LPS) was used to activate classically activated macrophages (M1 macrophages). LPS stimulation upregulated the expression of the CD86 antibody labeled with red fluorescence on macrophages, which was significantly decreased after co-incubation with the Ce–Aln gel (Fig. 3h). Similarly, the expression of the CD206 antibody labeled by green fluorescence was downregulated after the incubation with LPS and obviously increased after co-incubation with the Ce–Aln gel (Fig. 3h and Fig. S26). The Ce–Aln gel exhibited a strong capacity to modulate the macrophage phenotype when LPS stimulated the macrophages to express large amounts of CD86 protein, which mainly resulted from the coordinated Ce ions (Fig. S27). All these results revealed that the Ce–Aln gel reduced the ratio of M1 macrophages and increased the polarization of M2 macrophages.

Due to the multiple physiological functions of inflammatory cytokines in the microenvironment, the levels of inflammatory factors released by macrophages after various treatments were further evaluated by using enzyme-linked immunosorbent assay (ELISA) kits. LPS promotes the release of pro-inflammatory cytokines, such as interleukin-6 (IL-6) and tumor necrosis factor-α (TNF-α), which have a negative effect on fracture healing by stimulating osteocyte apoptosis, inducing the expression of RANKL and promoting osteoclast formation [8]. The levels of TNF-α and IL-6 were well mitigated after co-incubation with the Ce–Aln gel (Fig. 3i). An increase in M1 phenotype macrophages also led to a decrease in interleukin-4 (IL-4) and interleukin-10 (IL-10) levels. As a recognized inflammatory and immunosuppressive factor, IL-10 plays a key role in inflammation and bone healing [38]. Nonetheless, the Ce–Aln gel significantly upregulated the expression and release of IL-10 and IL-4 when macrophages were promoted to the M2 phenotype (Fig. S28). Notably, Aln alone had little effect compared with that of the control, demonstrating the necessity for the coordination of Ce–Aln. Therefore, the mechanism by which Ce–Aln gel regulates macrophage polarization was further explored. LPS activated the NF-κB pathway, while the Ce ions and Ce–Aln gel groups exhibited a decreased phosphorylation process of IκBα and the NF-κB p65 dimer (Fig. 3j and Fig. S29), indicating that the Ce–Aln gel regulated macrophage polarization through inhibition of the NF-κB pathway and that the inhibitory effect was mainly attributed to the presence of Ce ions. All the results revealed that coordination with Ce ions allowed the gel to acquire the ability to modulate the phenotype of macrophages. By inhibiting the activation of NF-κB pathways in M1 macrophages (Fig. 3k), the Ce–Aln gel effectively improved the inflammatory microenvironment by increasing the levels of anti-inflammatory factors and decreasing the levels of pro-inflammatory factors.

### Surface mineralization ability and bone metabolism regulation effect of the Ce–Aln gel

**Figure 4.**
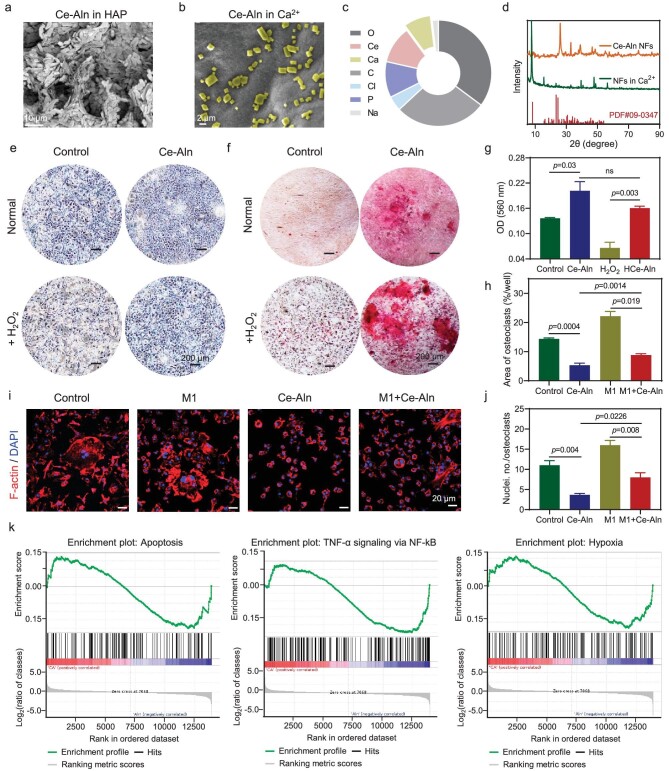
Bone metabolic regulation and RNA sequencing analysis of the Ce–Aln gel. (a) SEM image of the Ce–Aln gel after immersion in hydroxyapatite (HAP) for 6 h. Scale bar: 10 μm. (b) SEM image of the Ce–Aln gel after immersion in a calcium chloride solution for 3 h. (c) Statistical analysis of EDS mapping in (b) (*n* = 3). (d) XRD patterns of fibers before and after soaking in calcium ions (JCPDS.09–0347). (e) Photograph of MC3T3-E1 cells stained with alkaline phosphatase (ALP) for 7 days and (f) alizarin red S (ARS) for 14 days after different treatments. Scale bar: 200 μm. (g) Statistical analysis of the ARS staining in (f) (*n* = 3). (h) Statistical analysis of the TRAP staining areas in osteoclasts induced by mouse bone marrow mononuclear cells (BMMs) after different treatments (*n* = 3). (i) CLSM observation of osteoclasts after different treatments stained with phalloidin and DAPI. Scale bar: 20 μm. (j) Statistical analysis of the number of osteoclast nuclei in (i) (*n* = 3). (k) Gene set enrichment analysis (Aln vs CA). *P < 0.05; **P < 0.01; ***P < 0.001; ****P < 0.0001, determined by using Student's *t*-test; ns, not significant.

The presence of Ca^2+^/phosphate ions in scaffolds can facilitate the formation of an apatite layer on the surface of implants, thereby improving bone conduction and osteoinduction [39,40]. Hence, bone-like inorganic mineral hydroxyapatite (HAP) was soaked with Ce–Aln gel to measure the bone affinity of the scaffold. Compared with the alginate calcium hydrogel, the Ce–Aln gel adsorbed HAP from the surrounding environment and the adsorption amount was enhanced with increasing time (Fig. 4a and Fig. S30). XRD showed that a hexagonal HAP structure was present in the soaked gel (Fig. S31). When immersed in the calcium chloride solution, the phosphate in the gel combined with Ca^2+^ ions to form mineralized products of calcium (Fig. 4b) and the adsorption rate of the calcium ions was ∼8.22% compared with that of the untreated gel (Fig. 4c), further confirming that the Ce–Aln gel bound to endogenous Ca^2+^ and mineralized on the surface. Similarly, Ce–Aln NFs were immersed in a calcium chloride solution. XRD patterns and selected area electron diffraction of the mineralization products revealed that the mineralization products were mainly Ca(H_2_PO_4_)_2_ (Fig. S32 and Fig. 4d). Moreover, the increased Ca^2+^ in the environment did not affect the cells for a long time (Fig. S33).

Next, the effects of the Ce–Aln gel on the cells during bone remodeling were investigated. After various treatments, the alkaline phosphatase (ALP) expression and alizarin red S (ARS) staining of the MC3T3-E1 cells were analysed. The Ce–Aln gel promoted osteogenic differentiation with the increasing expression of ALP (Fig. 4e) and the generation of more calcium nodules (Fig. 4f), while H_2_O_2_ strongly inhibited osteogenic differentiation in a concentration-dependent manner (Fig. S34). Moreover, the suppressive effect of H_2_O_2_ was alleviated by the HCe–Aln gel (Fig. 4g), which was also verified by the relative activity of ALP extracted from cells (Fig. S35), implying that the process of osteogenesis was promoted by scavenging excess ROS from the microenvironment after Ce–Aln gel implantation. Moreover, compared with the H_2_O_2_ treatment group, Ce–Aln gel treatment significantly increased the expression of the osteogenic proteins osteopontin (OPN) and osteocalcin (OCN) in MC3T3-E1 cells, especially the protein expression of OPN, according to the WB results (Fig. S36). Furthermore, mouse bone marrow mononuclear cells (BMMs) were isolated for osteoclast induction. *In vitro* tartrate resistant acid phosphatase (TRAP) and cytoskeleton staining showed that the Ce–Aln gel efficaciously inhibited the emergence of osteoclasts (Fig. S37 and Fig. 4h). Subsequently, to mimic osteoclast formation under inflammatory conditions, the supernatant of RAW264.7 cells incubated with LPS was collected for further co-incubation with osteoclasts during induction (labeled the M1 group). More osteoclasts were formed in the M1 group, indicating that the release of macrophages in the pro-inflammatory state indeed promoted the formation of osteoclasts. However, the TRAP staining area and the number of osteoclast nuclei significantly decreased after incubation with the Ce–Aln gel (Fig. 4i and j), highlighting the osteoclast inhibitory capacity of the gel in an inflammatory environment. All these results indicated that the inflammatory microenvironment accelerated the inhibition of osteogenic differentiation and osteoclast formation, which was effectively reversed by the Ce–Aln gel through regulating the inflammatory microenvironment and restoring the balance of bone metabolism to promote bone formation.

### RNA sequencing reveals the mechanism of Ce–Aln gel promoting osteoblasts proliferation and differentiation

Subsequently, RNA sequencing was further applied to explore the regulation and potential mechanisms of the Ce–Aln gel on MC3T3-E1 cells. The volcano plot showed that the H_2_O_2_-plus-Ce–Aln gel-treated group (CA) displayed significant differences in gene expression compared with the H_2_O_2_-treated group (Con) and the CA group also showed significant differences compared with the H_2_O_2_-plus-Aln group (Aln), while the genes with significantly different expression in Con vs CA were similar to those in Aln vs CA, indicating that the osteogenic mechanism of the Ce–Aln gel under high ROS may be mainly derived from cerium ions (Fig. S38a and b). Moreover, the heat map showed that the CA group exhibited increased downregulation of the ‘Il6’ and ‘Nfkbib’ genes and upregulated the ‘Il4’, ‘Col6a1’ and ‘Bmp2’ genes (Fig. S38c), suggesting that the Ce–Aln gel promoted the growth of inorganic and organic components of bone tissue and antioxidant and anti-inflammatory regulation compared with Aln. To reveal the functions of the highly expressed differentially expressed genes, gene ontology (GO) analysis was performed. The results of the Aln and CA groups showed several GO categories related to cell behavior, oxidative stress and osteogenesis, such as ‘cell adhesion’, ‘antioxidant activity’ and ‘ossification’ (Fig. S38d). All these results suggested that the Ce–Aln gel promoted osteoblast adhesion and differentiation, reducing oxidative stress to enhance osteogenesis. Furthermore, gene set enrichment analysis was performed to determine the enrichment of expressed genes associated with metabolic pathways. The results revealed that the differentially expressed genes in the CA group were mainly related to ‘apoptosis’, which corresponded to the downregulated differential genes such as ‘Bcl 10’ and ‘Bax’ in the previous heat map. The release of Ce ions significantly downregulated ‘hypoxic’ metabolism by catalysing the generation of oxygen from ROS. Additionally, the downregulation of ‘TNF-α signaling via NF-κB’ enrichment also proved that the Ce–Aln gel inhibited related inflammatory factors and inflammatory pathways (Fig. 4k). Therefore, the transcriptome sequencing results indicated that the Ce–Aln gel effectively reversed oxidative stress and ameliorated apoptosis, promoting the proliferation and differentiation of MC3T3-E1 cells by modulating multiple metabolic pathways.

### Therapeutic evaluation of Ce–Aln gel in a skull defect model of osteoporosis

**Figure 5.**
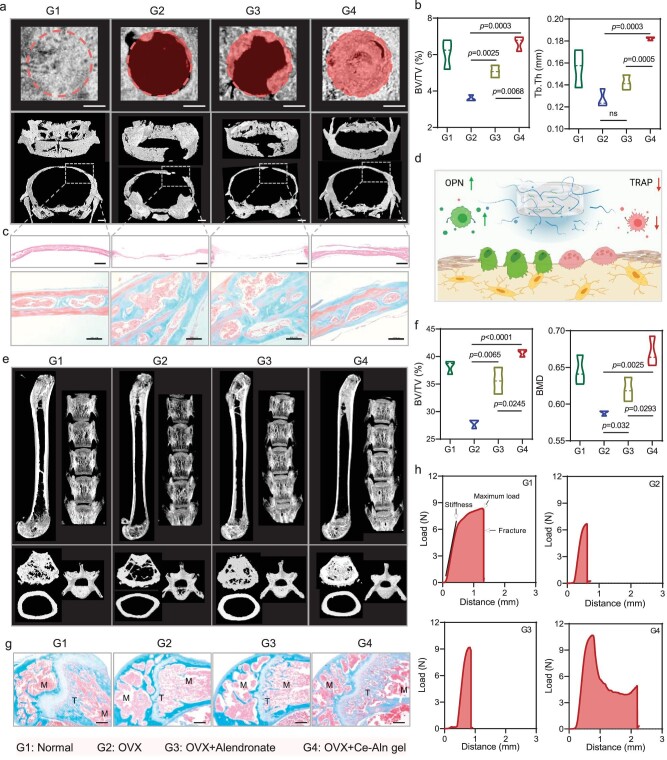
Therapeutic evaluation of the Ce–Aln gel in osteoporotic mice with skull defects. (a) Micro-CT images of the skulls of mice subjected to different treatments and 3D model images of the region of interest (ROI). Scale bar: 1 mm. (b) Statistical analysis of the relevant bone parameters of the 3D model of the ROI in (a) (*n* = 3). (c) Hematoxylin-eosin (H&E) and Masson tissue sections of the skulls from various groups. The former scale bar: 300 μm. The later scale bar: 100 μm. (d) Schematic diagram of the bone tissue repair through alleviating inflammation and regulating bone metabolism by the Ce–Aln gel (created with BioRender.com). (e) Micro-CT images of the spine and femur of mice subjected to different treatments and 3D model images of the ROI. (f) Statistical analysis of the relevant bone parameters of the 3D model of the ROI in (e) (*n* = 3). (g) Masson staining of tissue sections of the femurs from various groups. M: marrow, T: trabecula. Scale bar: 300 μm. (h) The mechanical curves of femurs from different groups of mice (*n* = 3). *P < 0.05; **P < 0.01; ***P < 0.001; ****P < 0.0001, as determined by using Student's *t*-test; ns, not significant.

To further verify the *in vivo* reversal effect of the Ce–Aln gel on bone repair in an osteoporotic environment, ovariectomized mice with a skull defect model were constructed. The mice without any treatment were used as the healthy control mice and were named the G1 group. After various treatments, the mice were sacrificed and the relevant tissues were removed for subsequent evaluation. Micro-computed tomography (micro-CT) scanning images of the mouse skull and 3D modeling images of the selected region were obtained (Fig. 5a). Compared with the obvious defect in the OVX group (G2), both the Aln (G3) and Ce–Aln gel (G4) groups promoted the growth of bone tissue in the defect. However, the proportion of the defect recovery area in the G4 group reached ∼90% (Fig. S39), which was much greater than that in the G3 group (∼40%). Furthermore, the quantitative analysis of the defect bone parameters revealed that the G4 group possessed a significantly higher bone volume fraction (BV/TV), bone mineral density (BMD), bone-surface-area-to-tissue-volume ratio (BS/TV), trabecular number (Tb.N), trabecular thickness (Tb.Th) and structural pattern index (SMI) than did the G2 group and some parameters were even obviously greater than those in the G3 group (Fig. 5b and Fig. S40). Next, hematoxylin-eosin (H&E) staining showed that the bone tissue in the G2 group grew slowly and that the defect was only fibrous tissue. In the G3 group, there was obvious bone growth at both ends of the defect; however, the bone still did not fully grow with some defects. Only the G4 group showed integrated bone tissue and bone lacuna at the defect site, which was similar to that observed in the healthy mice (Fig. 5c). After Masson staining of the bone tissues, the collagen of the new bone was mostly blue–green, while the collagen of the mature bone was red. Therefore, Masson staining revealed that the amount of red collagen in the G4 group was significantly greater than that in the G2 and G3 groups (Fig. S41). Immunohistochemical staining revealed that the G4 group exhibited largely increased expression of the osteogenic marker OPN (Fig. S42a) and the proportion of the OPN-positive region in the G4 group was 1.27 times greater than that in the G2 group (Fig. S42b). Compared with those in the G2 group, the number of osteoclasts (TRAP-positive cells) in the bone tissue was significantly lower after Aln and Ce–Aln gel treatments (Fig. S43), indicating that the Ce–Aln gel regulated bone metabolism to promote the process of bone remodeling. However, almost all relevant evaluations of the G4 group showed a much better therapeutic effect than that of the G3 group, which was ascribed to the ability of the Ce–Aln gel to regulate the inflammatory microenvironment to maintain bone reconstruction and achieve a better effect. Therefore, the NF-κB pathway was found to be activated in skull coloboma with osteoporosis after 1 week. However, the implantation of the Ce–Aln gel greatly inhibited the downregulation of the levels of the phosphorylated IκBα and NF-κB p65 proteins (Fig. S44), thereby reducing the continuous occurrence and spread of inflammation, which was undoubtedly beneficial to bone growth.

The above results confirmed that the Ce–Aln gel had a good therapeutic effect on local bone defects; however, the systemic osteoporosis was the main cause of the multiple fractures. Therefore, we evaluated whether the Ce–Aln gel had a positive impact on systemic osteoporosis in mice. Micro-CT scanning results of mouse femurs and spines revealed that the G2 group exhibited classic osteoporosis after ovariectomy, with bone volume reduction in the axial and accessory bones, and bone loss in the trabecular and cortical bones. However, this effect was ameliorated by the injection of Aln (G3) and the cancellous bone architecture and cortical bone thickness of the femurs and spines were greater in the G3 group (Fig. 5e), which was consistent with findings of clinical trials. Surprisingly, the bone loss in the femurs and spines was largely alleviated in the G4 group by the implanted Ce–Aln gel. Further analysis of bone parameters revealed that the gel-implanted mice exhibited increased BV/TV, BMD and BS/TV (Fig. 5f and Fig. S45), indicating significantly increased bone mass and bone microstructure of the total body. In addition, the H&E- and Masson-stained sections of the femoral ankle were presented. The porous loose bone and dissolved trabecular bone structure in the G2 group were relieved after treatment with Aln and Ce–Aln gel, and even a dense collagen fiber network and more trabecular bone microstructure appeared in the G4 group (Fig. S46 and Fig. 5g). Moreover, the biomechanical test of mouse femurs showed that the femurs in the Ce–Aln gel group withstood greater mechanical loads and had greater stiffness than those in the OVX and Aln groups (Fig. S47a and Fig. 5h). The statistical results displayed that the maximum load in the Ce–Aln gel group was ∼1.74 times greater than that in the OVX group and ∼1.15 times greater than that in the Aln group. The femur stiffness of Ce–Aln gel-treated mice was ∼2.62 times greater than that of OVX mice and ∼1.24 times greater than that of Aln mice (Fig. S47b and c), which further confirmed the therapeutic effect of the Ce–Aln gel on osteoporotic mice. Furthermore, TRAP staining showed that osteoclasts (black arrows) at the edge of the bone lacuna led to the loss of bone trabeculae and the dissolution of bone scale lines in the G2 group, while this phenomenon was significantly reduced in the G3 and G4 groups, and the G4 group exhibited more intact bone scale lines (Fig. S48). Furthermore, the evaluation of cytokines in the serum of the mice showed that the Ce–Aln gel decreased the levels of pro-inflammatory factors, such as TNF-α and IL-1β, and increased the levels of anti-inflammatory factors, such as IL-10 and IL-4, indicating that the locally implanted Ce–Aln gel further modulated the systemic inflammatory microenvironment (Fig. S49). Owing to the observation that the gel changed after ROS kindling, the Ce–Aln gel similarly degraded in the presence of ROS and transformed from fibrous ligands into nanodots *in vitro*. Therefore, it may circulate throughout the whole body and increase the bioavailability of Aln. Considering the rapid release rate of the Ce–Aln gel in the early stage, the concentration of Ce ions in mouse plasma was detected at Week 1 after implantation. The concentration of Ce ions was ∼0.2 mM in plasma after the implantation of the Ce–Aln gel and four times greater than that of the untreated group (Fig. S50), which indicated that the Ce–Aln gel did not cause high levels of Ce ions in the blood. Overall, the Ce–Aln gel effectively promoted bone tissue at the defect site by inhibiting osteoclasts and accelerating osteogenic transformation (Fig. 5d). In addition, the Ce–Aln gel also exhibited an outstanding therapeutic effect on systemic osteoporosis and ameliorated bone mass and microstructure, highlighting that it is a promising scaffold for osteoporotic bone repair.

## CONCLUSION

In summary, the problem of osteoporosis is not only an imbalance in bone metabolism, but also a dilemma of easy fracture but difficulty in recovery. Autocatalytic bifunctional metal supramolecular hydrogels based on clinical anti-osteoporosis drugs were constructed for the treatment of osteoporotic bone injury. With good viscoelasticity and injectability, the obtained Ce–Aln gel with a pH response was self-assembled from Aln and Ce ions, and it exhibited autocatalytic amplification of antioxidant capacity in response to ROS to further enhance the ROS-scavenging effects. *In vitro* experiments showed that the Ce–Aln gel protected cells from damage by scavenging excessive ROS to promote the recovery of normal physiological functions. Moreover, the Ce–Aln gel regulated the polarization phenotype of macrophages by inhibiting the activated NF-κB pathway and promoted the release of anti-inflammatory factors and the downregulation of pro-inflammatory factors, thereby improving the inflammatory microenvironment. Additionally, RNA sequencing revealed proof that the apoptosis signaling, hypoxia metabolism and TNF-α signaling via NF-κB that were related to immunity were significantly downregulated in the Ce–Aln gel-treated group compared with those in the Aln group. By inhibiting osteoclast activity via Aln, the Ce–Aln gel further ameliorated the imbalance in bone metabolism and effectively promoted osteogenic differentiation. *In vivo* experiments showed that the inflammatory microenvironment at the defect was improved and the bone tissue repair was significantly enhanced after implantation of Ce–Aln gel into the skull defects of OVX mice. More excitingly, systemic osteoporosis was greatly ameliorated in OVX mice, as indicated by significantly enhanced bone mass and microstructure. Therefore, this clinical drug–metal coordination gel is an ideal biological carrier-free drug platform for treating osteoporosis and related bone repair in patients with imbalanced bone metabolism.

## ETHICAL STATEMENTS

The animal experiments in this study were carried out according to the laboratory animal welfare Chinese National Standard (GB/T 35892–2018) and approved by the Laboratory Animal Center of Soochow University (No. ECSU-202107A0270).

## Supplementary Material

nwae209_Supplemental_File
