# Supplementary material for: Autocatalytic bifunctional supramolecular hydrogels for osteoporotic bone repair
Source: Natl Sci Rev. 2024 Jun 20;11(7):nwae209. doi: 10.1093/nsr/nwae209 (PMC11275467; doi:10.1093/nsr/nwae209)
Supplement: nwae209_Supplemental_File [file nwae209_supplemental_file.pdf]

Supporting Information

Autocatalytic bifunctional supramolecular hydrogels for osteoporotic bone repair

Zhihui Han[1], Xiang Gao[2], Yuanjie Wang[1], Cheng Huang[2], Hao Song[2], Shuning Cheng[1], Xiaoyuan Yang[1], Xiaoliang Cui[1], Jie Wu[1], Kailu Wei[1], Liang Cheng*[1,3]

[1]Institute of Functional Nano & Soft Materials (FUNSOM), Collaborative Innovation Center of Suzhou Nano Science and Technology, Soochow University, Suzhou, Jiangsu, 215123, China

[2]Department of Orthopedics, The Second Affiliated Hospital of Soochow University, Suzhou, Jiangsu 215004, China

[3]Macao Institute of Materials Science and Engineering, Macau University of Science and Technology, Taipa 999078, Macau SAR, China

Email: lcheng2@suda.edu.cn

1. Experiment Sections

Materials:

Cerium trichloride pentahydrate ($CeCl_3 \cdot 5H_2O$), alendronate, methanol (99%), hydrogen peroxide (H_2O_2), calcium chloride ($CaCl_2$), and ethylene diamine tetraacetic acid (EDTA) were obtained from Aladdin Industrial Co. Ltd. (Shanghai, China). Potassium persulfate ($K_2S_2O_8$), 2,2'-azinobis-(3-ethylbenzthiazoline-6-sulphonate) (ABTS), 3,3',5,5'-tetramethylbenzidine (TMB), sodium hydroxide (NaOH), hydroxyapatite (HAP), cetyl pyridinium chloride, and methyl blue (MB) were obtained from McLean Biochemical Technology Co. Ltd. (Shanghai, China). All chemicals were of analytical grade and used without further purification.

Preparation and Characterization of Ce-Aln NFs:

Ce-Aln nanofibers were prepared according to the following procedure. Cerium trichloride pentahydrate (1 mM) was dissolved in a serum flask containing double distilled water. Then, sodium alendronate (0.5 mM) was added, and the mixture was stirred continuously for 3 h at room temperature. During the process, the solution changed from colorless to viscous white. After that, the solution was centrifuged at 14800 rpm for 5 mins, followed by washing with water for three times. The above specimens were observed and characterized by transmission electron microscopy (TEM, FEI Tecnai F20, Oregon, USA) with energy dispersive spectrometer (EDS) and Fourier transform infrared spectrometer (FT-IR, V70 & Hyperion 1000, Bruker, Billerica, USA). The zeta potential was measured by dynamic light scattering (DLS) instrument (Malvern, Worcestershire, UK). X-ray photoelectron spectroscopy (XPS) analysis was performed using a PHI Quantera SXM X-ray photoelectron spectrometer with an Al Ka monochromator source (Kratos, Manchester, UK) to

determine the chemical valence states. Inductively coupled plasma optical emission spectrometry (ICP-OES) was applied to quantify the Ce in the Ce-Aln nanofibers.

Preparation and characterization of the Ce-Aln fibro-gel

The obtained Ce-Aln nanofibers were mixed with sodium hydroxide at various ratios. A rotated rheometer (DHR-2, TA, USA) was used to obtain the fibro-gels with higher mechanical strength. The morphology and elementary composition of the fibro-gels were observed by scanning electric microscopy (SEM, Zeiss, Germany) with EDS and TEM imaging. FT-IR spectra showed the structure and bonding, while the XPS analysis revealed the valence of Ce. To verify the injectability and dynamic reversibility of the Ce-Aln gel, a methyl blue (MB) probe was added to NaOH to obtain a colorful gel.

Free radical scavenging and antioxidant tests

The ABTS and ox-TMB probes were applied to detect the antioxidant capacity of Ce-Aln for $ABTS^{+\cdot}$ and ·OH, respectively. In detail, $ABTS^{+\cdot}$ was obtained by incubating 0.8 mL ABTS (4 mg/mL) and 1 mL of potassium persulfate ($K_2S_2O_8$, 1 mg/mL) overnight in the dark. ~ 40 μL of $ABTS^{+\cdot}$ was added into different amounts of Ce-Aln NFs. Then, the absorbance values of $ABTS^{+\cdot}$ at ~738 nm in the mixed solution were recorded to quantify the scavenging ability. Similarly, 5 μL of TMB, ~50 μL of H_2O_2 (10 mol/L), and ~50 μL of $FeCl_2$ (2 mg/mL) were mixed to obtain the ox-TMB probe, and then different amounts of Ce-Aln NFs were added. With the removal of ·OH, there was a change in the absorption peak at ~620 nm. The changes in the absorption spectra with time in the range of 300-1000 nm were determined. The changes in the absorbance at 517/738/620 nm monitored

by the ultraviolet and visible (UV-Vis) spectrophotometer (GENESYS 140, Thermo Fisher Scientific, USA).

Measurement of the oxygen production of the Ce-Aln gel in solution

The quantified Ce-Aln gel was placed in a 50 mL centrifuge tube containing 10 mL of double distilled water (DD H_2O). The detector of the oxygen dissolving meter was immersed in the DD H_2O, and the same amount of NaOH with different concentrations was added to detect the change in oxygen content with time.

***In vitro* degradation and release experiments**

The Ce-Aln gels were synthesized into 5 mm diameter and 3 mm height cylinders, accurately weighed (recorded as M_0), placed into 5 mL plastic tubes containing 4 mL of different solutions (PBS, DMEM, and PBS with H_2O_2), and then placed into a shaker at 120 rpm, 37°C. Samples were collected at each set time point and dried to constant weight (recorded as M_1), and weight loss (%) was calculated using the following formula:

$$\text{weight loss (\%)} = \frac{M_0 - M_1}{M_0} \times 100\% \quad (1)$$

The preliminary preparation for the release experiment was similar to that for degradation. However, the solutions were centrifuged at the set time points, and 10 μL of the supernatant was taken as the sample to be tested. The sample was mixed with aqua regia, heated, dissolved, and thoroughly, and then the volume was adjusted to 10 mL. After filtration, the concentration of Ce ions was detected

by an inductively coupled plasma optical emission spectrometer (ICP-OES, PerkinElmer, Waltham, USA).

Detection of porosity, swelling, and water retention:

Porosity: Ce-Aln gels were synthesized into cylinders with a radius (R) of 2.5 mm and a thickness (H) of 3 mm, and accurately measured (recorded as W_1). Then, the gels were immersed in ethanol for 24 h, and the wet weight was measured (recorded as W_2). Then the porosity (%) was calculated using the following formula:

$$\text{porosity (\%)} = \frac{W_2 - W_1}{\rho \cdot \pi R^2 \cdot H} \times 100\% \tag{2}$$

***Swelling rate*:** Ce-Aln gels of the above size were immersed in DD H_2O for 24 h at room temperature, then the surface water was subsequently wiped with filter paper and weighed (recorded as W_s), and the dry weight of the gel was recorded as W_d. Then the equilibrium swelling rate was calculated using the following formula:

$$\text{swelling rate (degree)} = \frac{W_s}{W_d} \tag{3}$$

Rheological detection of Ce-Aln gel

A rotational rheometer was used to measure the rheological properties of the self-assembled fiber gel. The plate spacing was set at 1 mm, and the test temperature was room temperature.

(1) The time-scan oscillation test was performed at a frequency of 1 Hz, 0.1% strain, and 100 s. The gel point was the time when the storage modulus (G ') exceeded the loss modulus (G '').

(2) The shear strain was set at 0.1%, and the angular frequency was varied from 0.1 rad/s to 100 rad/s. The storage modulus G 'and loss modulus G' of the gel were measured at different frequencies.

***In vitro* cellular experiments**

Mouse mononuclear macrophage leukemia cells (RAW 264.7) and mouse embryo osteoblast precursor cells (MC3T3-E1) were purchased from American Type Culture Collection (ATCC) and cultured in standard cell culture medium at 37 °C under 5% CO_2.

***In vitro* cell experiments of Ce-Aln hydrogels**

Cytotoxicity: MC3T3-E1 and RAW264.7 cells were seeded in 96-well plates at $1*10^4$ cells per well. The Ce-Aln gels prepared in a sterile environment were incubated with cells after being immersed in PBS for 24 h. The medium was changed every two days, and after 24 h of treatment, the cell viability was measured by the standard MTT assay, and the characteristic absorbance at ~ 490 nm was measured by a microplate reader (Bio tek, USA).

Cell proliferation: RAW264.7 cells were seeded in 96-well plates at 1×10^4 cells per well. The sterile gels were incubated with the cells after immersion. The medium was changed every two days, and after 1, 3, and 7 days of treatment, the cell viability was measured by the standard MTT assay, and the characteristic absorbance at ~ 490 nm was measured by a microplate reader. Similarly, MC3T3-E1 cells were seeded in small culture dishes at a density of 1×10^5 per dish. The sterile gels were incubated with the cells after immersion. The medium was replaced every two days, and after 1, 3, and 7 days of treatment, Calcein AM staining was performed, and the cells were observed under a confocal laser scanning microscope (CLSM, Zeiss Axio-Imager LSM-800).

Cytoskeleton staining: MC3T3-E1 cells were seeded in 24-well plates with cell slides at 5×10^4 cells per well. The soaked gels, H_2O_2 (200 μM), H_2O_2 plus Ce^{3+}, H_2O_2 plus Aln, and H_2O_2 plus Ce-Aln soaked gels were incubated with the cells for 8 h, respectively. The cells were stained with Alexa Fluor 647 phalloidin and observed under a confocal laser scanning microscope (CLSM, Zeiss Axio-Imager LSM-800).

Cellular ROS-eliminating experiments: MC3T3-E1 and RAW 264.7 cells were seeded in 24-well plates with cell slides at 5×10^4 cells per well. Then, the cells were either left untreated or treated with H_2O_2 (200 μM), H_2O_2 plus Ce^{3+}, H_2O_2 plus Aln, or H_2O_2 plus Ce-Aln soaked gels for 8 h. A 2',7'-dichlorodihydrofluorescein diacetate (DCFH-DA) probe was applied to stain the intracellular ROS for 30 mins. Then, the levels of ROS were observed through a confocal laser scanning microscope and the fluorescence was also analyzed using flow cytometry (C6 plus, Becton, Dickinson, and Company, USA) respectively.

Mitochondrial membrane potential staining: MC3T3-E1 cells were seeded in 24-well plates at a density of 5×10^4 cells per well. The soaked gels, H_2O_2 (200 μM), H_2O_2 plus Ce^{3+}, H_2O_2 plus Aln, and H_2O_2 plus Ce-Aln gels were incubated with the cells for 8 h, respectively. The MMP of the cells were determined by staining with 20 μM JC-1 for 20 mins. The cells were then washed with 1xPBS, and the fluorescence was analyzed using a confocal laser scanning microscope.

***In vitro* polarization of macrophages and inflammatory factor evaluation:** RAW 264.7 cells were seeded in 24-well plates with cell slides at 5×10^4 cells per well and incubated for 24 h. Then, these

cells were either untreated or treated with lipopolysaccharide (LPS, 100 ng/mL), LPS plus Ce^{3+}, LPS plus Aln, or LPS plus Ce-Aln soaked gels for 8 h. The M1 and M2 macrophages were labeled with CD86 and CD206, respectively. The fluorescence was analyzed using flow cytometry. Similarly, the polarization of macrophages was also evaluated by a confocal imaging.

Enzyme-linked immuno sorbent assay:

The supernatant culture solution from the above experiments was collected and analyzed. The levels of tumor necrosis factor-α (TNF-α), interleukin-6 (IL-6), interleukin-1β (IL-1β), interleukin-4 (IL-4), and interleukin-10 (IL-10) were quantified by ELISA kits (Invitrogen, USA) in accordance with the manufacturer's instructions.

Western blotting analysis: RAW 264.7 cells were seeded in 6-well plates at a at 1×10^5 cells per well and incubated for 24 h. Then, the cells were either untreated or treated with lipopolysaccharide (LPS, 100 ng/mL), LPS plus Ce^{3+}, LPS plus Aln, or LPS plus Ce-Aln soaked gels for 8 h. Then, the cells were lysed, and protease and phosphatase inhibitors were added to extract the protein. An enhanced BCA protein assay kit (Beyotime, Shanghai) was used to measure the protein concentration. Then, the proteins were mixed with SDS-loading buffer, boiled at 95 °C for 3 min for denaturation, and loaded on 12% or 10% (w/v) sodium dodecyl sulfate polyacrylamide gels. After the electrophoresis at 90 V, the proteins were transferred to nitrocellulose membranes at 200 A. The membranes were blocked with 5% BSA solution for 2 h at room temperature to avoid non-specific binding. Next, the membranes were probed with primary antibodies: Iκb, p-IκB, NF-κB p65, NF-κB p-p65, and β-actin (Proteintech, USA) overnight at 4 °C. Then, the membranes were washed with PBST 3 times and

reacted with HRP-labeled secondary antibodies for 60 mins another day. After washing, the membranes were developed using a chemiluminescence detection system. The final membranes were visualized using a DNA electrophoresis gel imager (AI600, General Electric, USA).

Bone affinity and calcium ion adsorption assay for the Ce-Aln gel

The affinity of Ce-Aln gel for bone minerals was tested and compared with that of calcium alginate gel. The selected gels were all cylinders with a diameter of 3 mm. Hydroxyapatite (HAP) was incubated with the same volume of gels in a microcentrifuge tube for various time (1.5, 3, and 6 h). After centrifugation by centrifugation, the supernatant was removed, and the gels were washed several times with deionized water, then lyophilized, and characterized by SEM.

Similarly, the gels of the same size were immersed in calcium chloride solution (1 mg mL^{-1}) at room temperature for various time (0.5, 1.5, and 3 h). Then, the supernatant was aspirated to determine the concentration of Ca ions by ICP-OES. Meanwhile, the gel was washed several times with deionized water, and the surface was observed by SEM after lyophilization.

Osteogenic differentiation and evaluation

In the osteogenic induction differentiation assay, alkaline phosphatase (ALP) activity and alizarin red S (ARS, Beyotime, Shanghai) were used to evaluate the degree of mineralization, respectively. MC3T3-E1 cells were seeded in 12-well plates at a density of $1*10^5$ / well, and 1 mL of special osteogenic differentiation induction solution was added. Then, the cells were treated with hydrogen peroxide (H_2O_2, 200 μM), Ce-Aln gel, and H_2O_2 plus Ce-Aln gel, respectively. Osteogenic induction medium alone served as the control. The solution was replaced every two days. After 7 days of

treatment, an ALP detection kit (Solarbio, Beijing) was used for staining. The stained cells were observed under a light microscope (Leica, Germany) and photographed. Moreover, ALP activity in the cells was detected with an ALP activity detection kit (Beyotime, Shanghai), and the quantitative analysis was performed by a microplate reader. Similarly, the apoptosis-related protein Bcl2-associated X (Bax), osteopontin (OPN) and osteocalcin (OCN) protein expression changes of osteoblasts after different treatment were analyzed by WB analysis.

After 14 days of the treatment, a solution of ARS was applied for staining. The stained cells were also observed under a light microscope and photographed. A 10% (w/v) aqueous solution of cetyl pyridinium chloride was added to each well to quantify calcium salt deposition in each group of cells. Visible light absorption at ~ 562 nm was determined by a microplate reader.

Osteoclast induction and evaluation

4 to 6 weeks-old C57bl/6 female mice were aseptically treated and sacrificed, after which the bone marrow monocytes (BMMs) in mouse femurs were extracted and cultured in large dishes with minimum essential medium α (αMEM, Gibico, USA). The suspended cells in the supernatant were removed 14 to 16 h later, seeded in plates, and treated with 30 ng mL^{-1} macrophage colony stimulating factor (M-CSF, R&D). After 5 days of culture, the cells were treated with 50 ng mL^{-1} receptor activator of nuclear factor kappa-B ligand (RANKL, R&D) of and 30 ng mL^{-1} M-CSF. The solution was replaced every two days, and the induction lasted for about 12 days. To evaluate the effect of Ce-Aln gel on osteoclasts, BMMs were treated with culture medium from RAW264.7 cells after LPS induction (M1), Ce-Aln gel, and M1 plus Ce-Aln soaked gels supplemented with M-CSF and RANKL. Then, the cytoskeleton of the treated cells were stained for fibros actin (F-actin) using Alexa

Fluor 647 phalloidin, and the nuclei were stained with 4,6 -diamidino- 2-phenylindole (DAPI) to observe the occurrence of osteoclasts. For quantitative analysis, the cells with more than three nuclei observed were counted as osteoclasts. The tartrate-resistant acid phosphatase (TRAP) staining of cells was performed with a tartrate-resistant acid phosphatase staining kits (Jiancheng, Nanjing).

mRNA library construction and sequencing

Total RNA was extracted from cells using TRIzol reagent (Invitrogen, Carlsbad, CA, USA) according to the vendor's instruction. Total RNA (1 μg) was used for subsequent library preparation. Poly(A) mRNA isolation was performed using oligo(dT) beads. The mRNA fragmentation was performed using divalent cations and high temperature. Priming was performed using random primers. First strand cDNA and second-strand cDNA were synthesized. The purified double-stranded cDNA was then treated to repair both ends, and a dA-tail was added in one reaction, followed by a T-A ligation to add adaptors to both ends. Size selection of the adaptor-ligated DNA was then performed using DNA clean beads. Each sample was then amplified by PCR using the P5 and P7 primers and the PCR products were validated. Then, libraries with different indices were multiplexed and loaded on an Illumina HiSeq/ Illumina Novaseq/ MGI2000 instrument for sequencing using a 2x150 paired-end (PE) configuration according to vendor's instructions.

Surgical procedure

Female C57bl/6 mice aged 6-8 weeks were purchased from Changzhou Cavins Biological Technology Co., Ltd., China. All experimental procedures were performed according to protocols approved by Laboratory Animal Center of Soochow University. To establish the osteoporotic mouse

model, OVX mice were subjected ovariectomy. The success of the osteoporosis model was confirmed by continuous observation of body weight changes. After 6 weeks, the OVX mice were anesthetized, and the cranial region was sterilized. A slow diamond drill was used to create a 3 mm diameter defect on the left side of the skull. Fifteen OVX mice were randomly divided into three groups: the OVX group (no material implanted after skull modeling), the Aln group (alendronate of 2 ug was injected via tail vein every week after skull modeling), and the Ce-Aln group (Ce-Aln gel implanted after skull modeling). The skin layer was closed with a 6-0 suture.

Ce ions detection in plasma

The OVX mice were implanted with Ce-Aln gel in the skull defect, and after 7 days of treatment, the blood of the mice was obtained by removing the eyeball and placing it in an anticoagulant tube, standing on ice for 20 min. Then the blood was centrifuge at 2500 rpm for 5 min, and then mouse plasma was obtained. Mouse plasma was collected and placed in a beaker, dissolved by aqua regia and treated at 300 °C. The obtained sample is tested by ICP-OES after the process of constant volume and filtration.

Histological analysis:

The skull and femur tissues of the sacrificed mice were immersed in EDTA solution for decalcification at 37°C for 14 days. The decalcified tissue was embedded in paraffin for tissue slice preparation and stained with H&E, Masson, TRAP, and OPN for histological assays, and then observed by a fluorescence optical microscope (Leica, Germany)

Statistical analysis:

All results are presented as the mean ± standard error of mean (SEM). All experiments were repeated at least three times. Each condition was analyzed in triplicate. $^{*}p < 0.05$, $^{**}p < 0.01$, and $^{***}p < 0.001$; n.s. represents no significant difference.

2. Supporting Figures

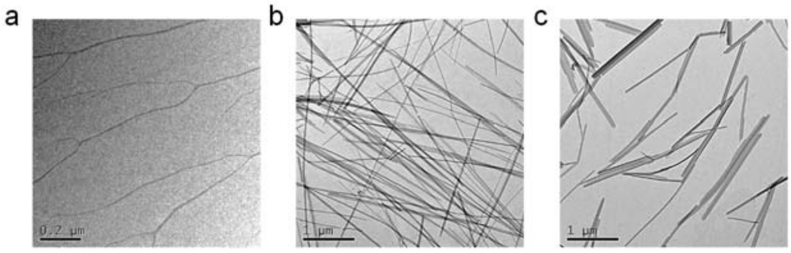

Figure S1. TEM images of Ce-Aln nanocomplexes at various ratios. (a) Ce-Aln nanocomplexes at a ratio of 2 : 1. (b) Ce-Aln nanocomplexes at a ratio of 1 : 1. (c) Ce-Aln nanocomplexes at a ratio of 1 : 2.

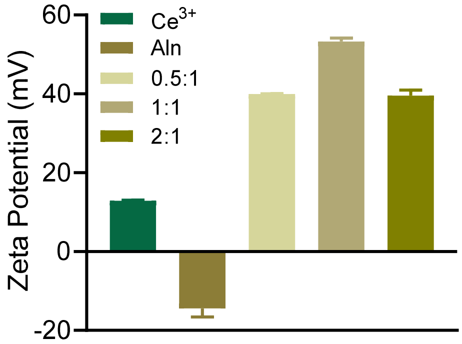

Figure S2. Zeta potentials of Ce^{3+}, alendronate (Aln), and Ce-Aln nanocomplexes at various ratios.

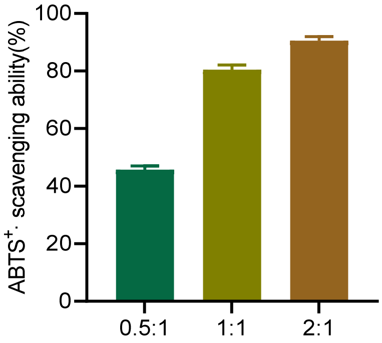

Figure S3. $ABTS^{+}\cdot$ scavenging ability of Ce-Aln nanocomplexes at various ratios (n = 3).

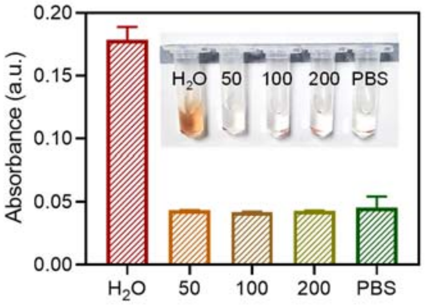

Figure S4. Quantitative analysis of the hemolysis assay of the Ce-Aln NFs. 1, 2, and 3 represent the gradual increase in the concentration of Ce-Aln NFs (50, 100, and 200 μg/mL, respectively. n = 3).

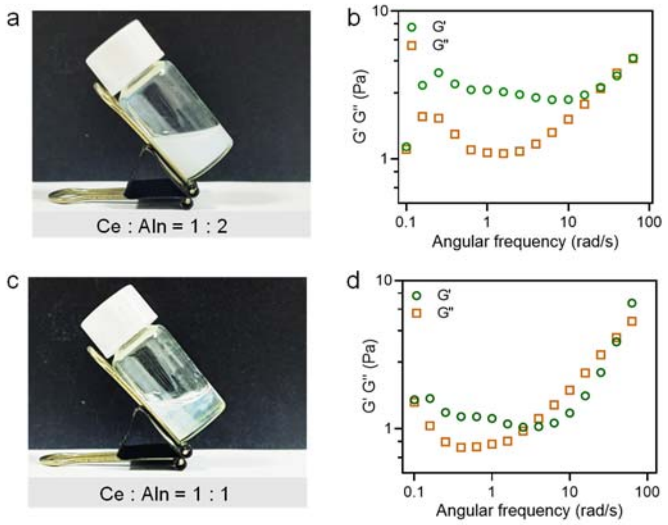

Figure S5. Detection of the pH-responsive self-assembled into gels of the Ce-Aln nanocomplexes at various proportions. (a) Photographs of the Ce-Aln nanocomplexes at a ratio of 1:2. (b) Angular frequency-scan curve for the rheological characterization of Ce-Aln nanocomplexes at a ratio of 1:2 (n = 3). (c) Photographs of the Ce-Aln nanocomplexes at a ratio of 1 : 1. (d) Angular frequency-scan curve for the rheological characterization of Ce-Aln nanocomplexes at a ratio of 1 : 1 (n = 3).

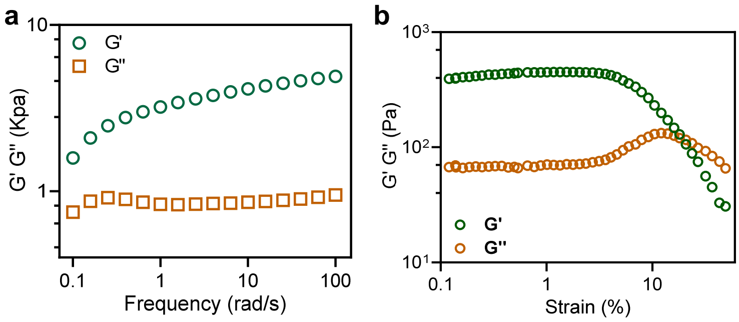

Figure S6. Characterization of the rheological properties of the Ce-Aln gel. (a) Angular frequency scan curve of Ce-Aln gel (b) Oscillation strain-scan curve from the rheological characterization of the Ce-Aln gel. The crossover point occurred at a strain of 19%.

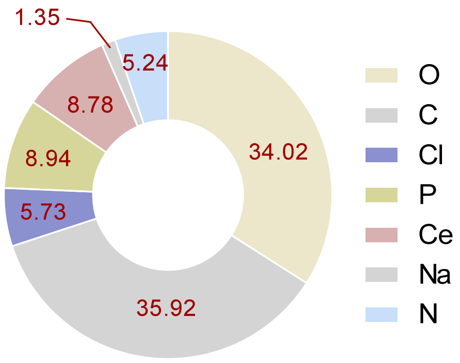

Figure S7. Quantitative analysis of EDS mapping of the Ce-Aln gel.

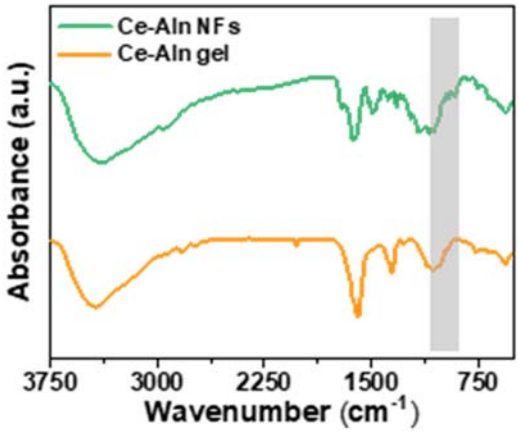

Figure S8. FT-IR spectra of the Ce-Aln NFs and Ce-Aln gel.

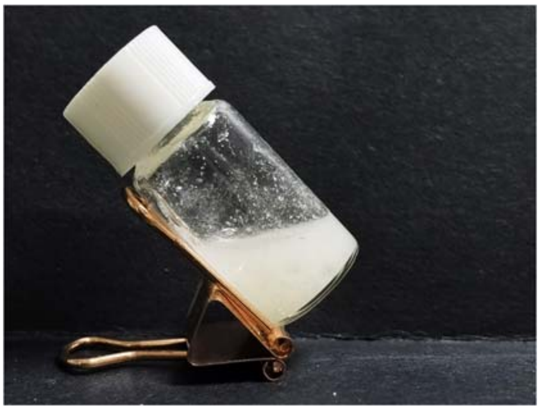

Figure S9. Photograph of the Ce-Aln gel after EDTA treatment.

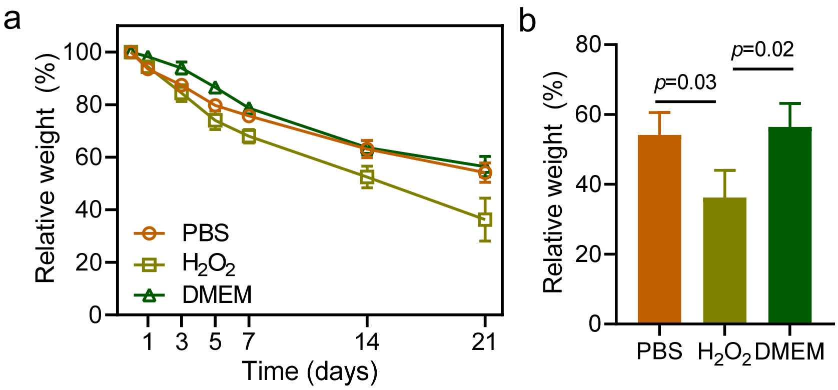

Figure S10. *In vitro* evaluation of Ce-Aln gel degradation. (a) Relative weight of the Ce-Aln gel in different physiological solutions (PBS, H_2O_2, DMEM) (n = 3). (b) Relative weight of the Ce-Aln gel in the various solutions on day 21.

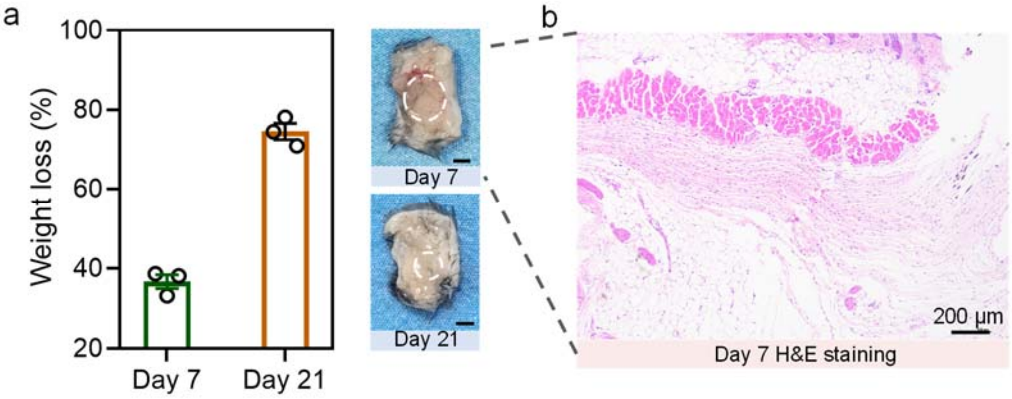

Figure S11. *In vivo* degradation evaluation of Ce-Aln gel. (a) Weight loss after implantation of the Ce-Aln gel for different time (n = 3). The right panels were skin tissue from the backs of mice after

Ce-Aln gel implantation for 7 days (up) or 21 days (bottom). (b) H&E staining of skin tissue after Ce-Aln gel implantation for 7 days.

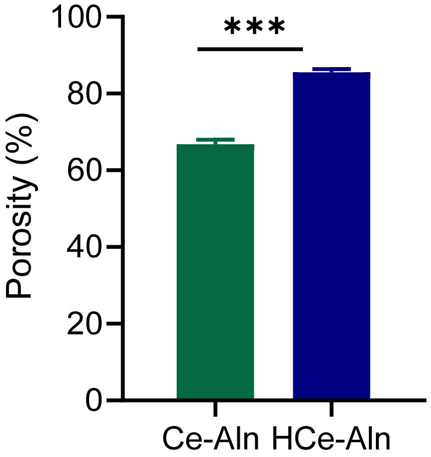

Figure S12. Porosity of the Ce-Aln gel and the Ce-Aln gel after pretreatment with H_2O_2 (HCe-Aln gel) (n = 3). **P* < 0.05, ***P* < 0.01, ****P* < 0.001, and *****P* < 0.0001, as determined by a Student's t-test; ns, not significant.

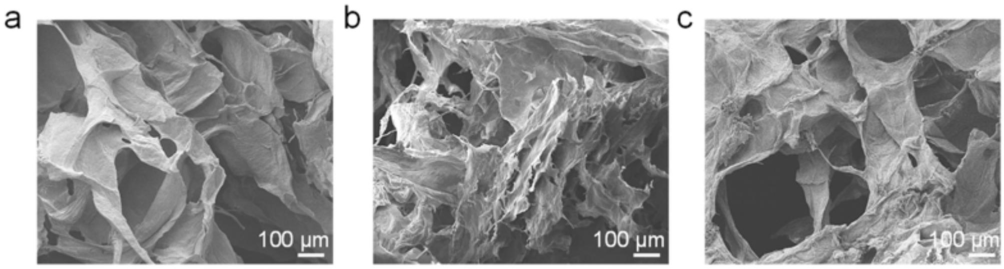

Figure S13. SEM image of the Ce-Aln gel after treatment with (a) H_2O_2, (b) $ABTS^{+}\cdot$, and (c) ·OH. Scale bar: 100 µm.

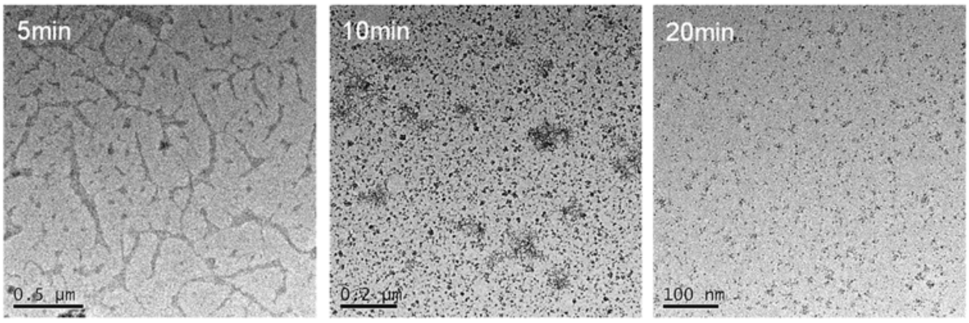

Figure S14. TEM images of the Ce-Aln gel after pretreatment with H_2O_2 for different time.

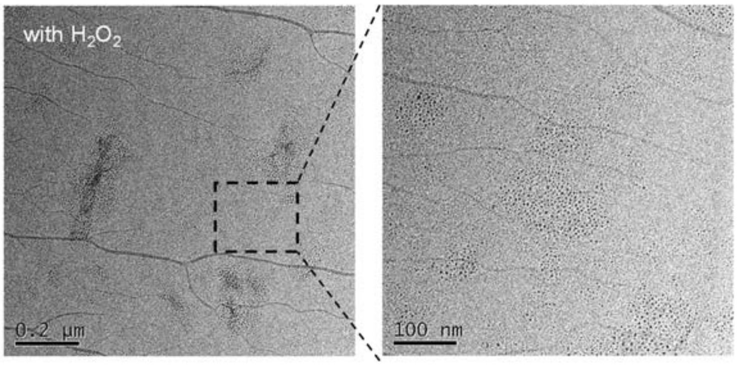

Figure S15. TEM images (left) and high-resolution TEM (HRTEM) images (right) of the Ce-Aln NFs pretreated with H_2O_2.

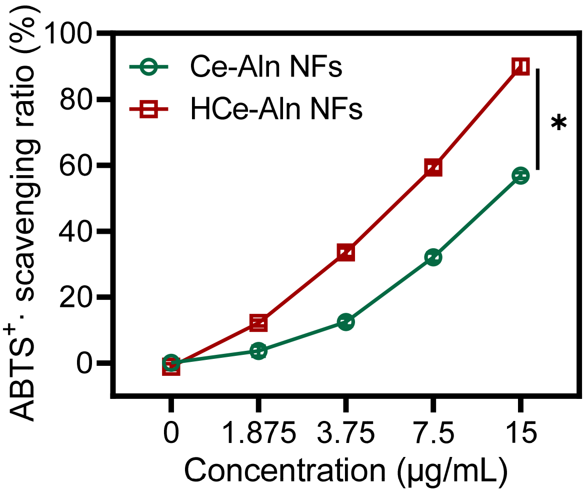

Figure S16. Quantification of the $ABTS^{+}\cdot$scavenging capacity of the Ce-Aln NFs with/without pretreatment of H_2O_2 (HCe-Aln NFs) with different concentrations (n = 3). *P < 0.05, **P < 0.01, ***P < 0.001, and ****P < 0.0001, as determined by a Student's t-test; ns, not significant.

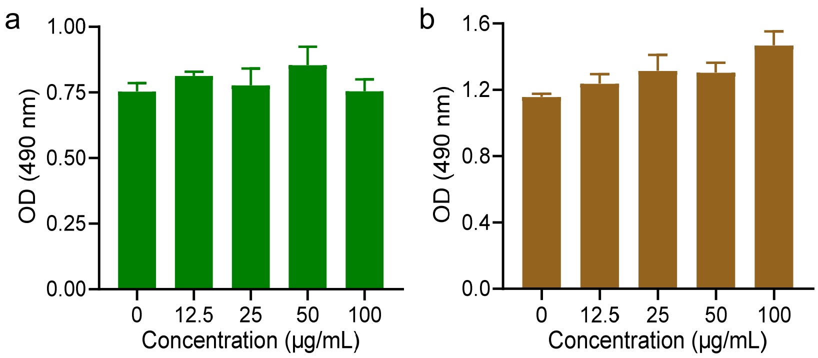

Figure S17. Cytocompatibility of the Ce-Aln gel for 24 h. (a) The viability of MC3T3-E1 cells after

various treatments (n = 6). (b) The viability of RAW264.7 cells after various treatments (n = 6).

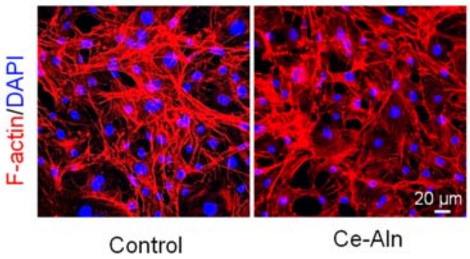

Figure S18. Confocal laser scanning microscopy (CLSM) of the cytoskeleton of MC3T3-E1 cells stained with phalloidin after different treatments.

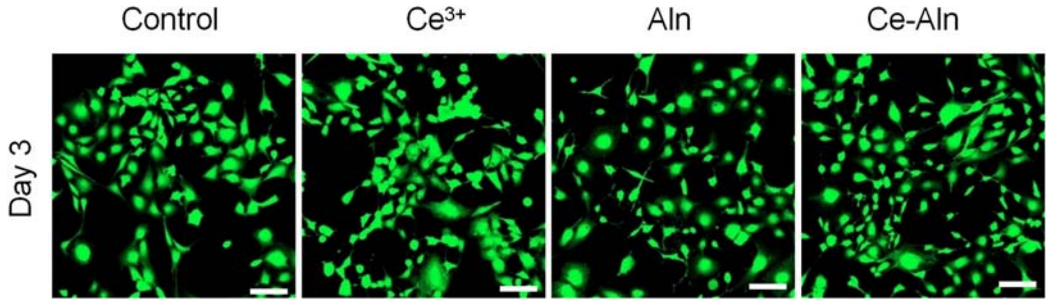

Figure S19. CLSM observation of MC3T3-E1 cells stained with Calcein AM at 3 days after various treatments. Scale bar: 100 μm.

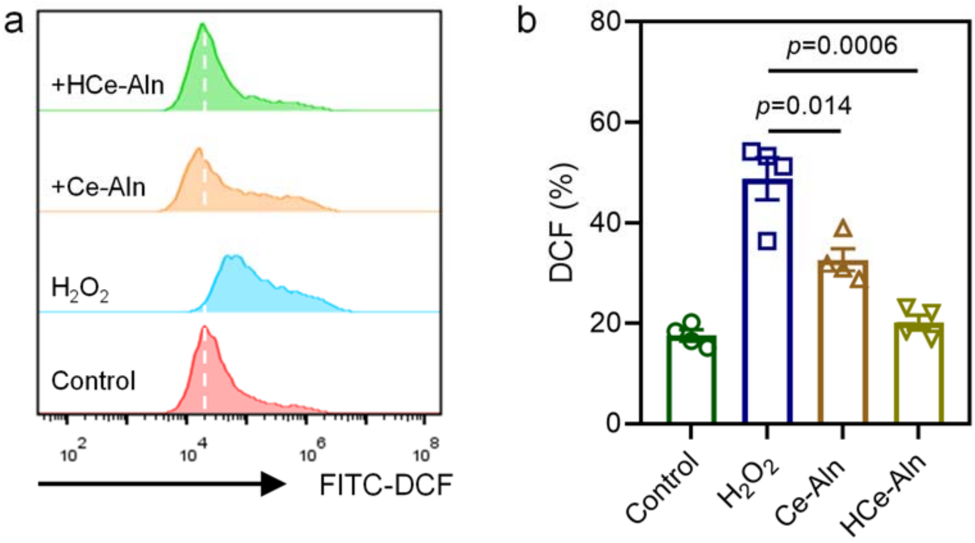

Figure S20. Evaluation of the ability of the Ce-Aln gel to clear ROS on RAW264.7 cells. (a) FACS

results showing intracellular ROS in RAW264.7 cells stained with DCFH-DA after various treatments. (b) Quantitative analysis of the FACs results of ROS/RNS levels in a (n = 4). **P* < 0.05, ***P* < 0.01, ****P* < 0.001, and *****P* < 0.0001, as determined by a Student's t-test; ns, not significant.

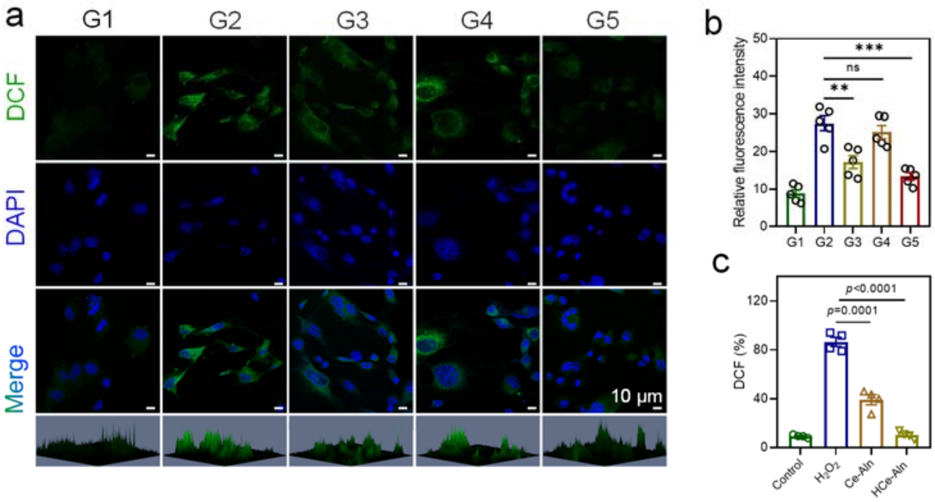

Figure S21. Evaluation of the ability of the Ce-Aln gel to clear ROS on MC3T3-E1 cells. (a) CLSM images of MC3T3-E1 cells after various treatments stained with DCFH-DA and DAPI. Scale bar: 10 μm; the bottom images show the corresponding fluorescence intensity of DCF. (b) Quantitative analysis of the relative fluorescence intensity of DCF in a (n = 4). (c) Quantitative analysis of the FACs results of ROS/RNS levels after various treatments stained with DCFH-DA (n = 4). (G1: Control; G2: H_2O_2; G3: H_2O_2 + Ce^{3+}; G4: H_2O_2 + Aln; G5: H_2O_2 + Ce-Aln). **P* < 0.05, ***P* < 0.01, ****P* < 0.001, and *****P* < 0.0001, as determined by a Student's t-test; ns, not significant.

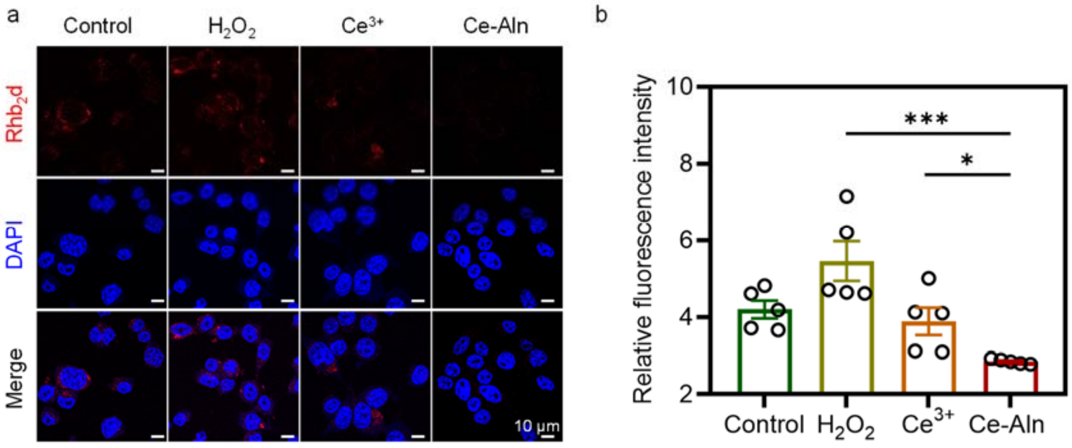

Figure S22. *In vitro* catalytic oxygen production of Ce-Aln gel. (a) CLSM observation of MC3T3-

E1 cells after various treatments stained with ruthenium tripyridine (Rhb2d). Scale bar: 10 μm. (b) Quantitative analysis of the relative fluorescence intensity of Rhb2d in a (n = 5). **P* < 0.05, ***P* < 0.01, ****P* < 0.001, and *****P* < 0.0001, as determined by Student's t-test; ns, not significant.

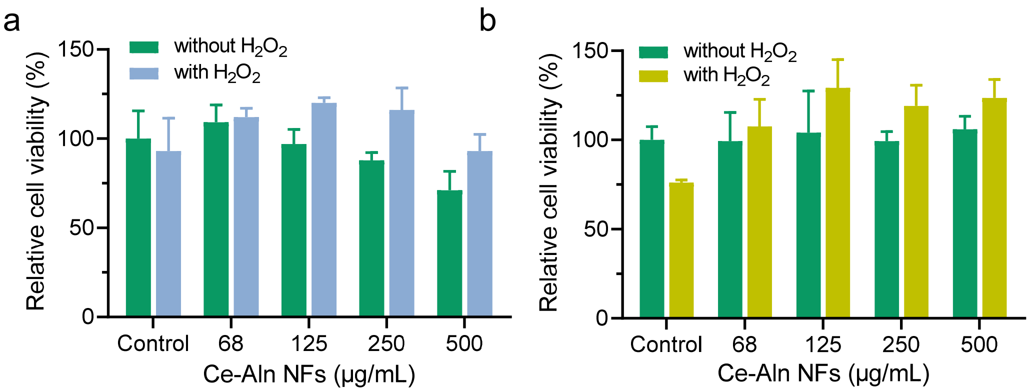

Figure S23. Cytocompatibility of the Ce-Aln NFs for 24 h. (a) The cell viability of MC3T3-E1 cells after various treatments (n = 6). (b) The cell viability of RAW264.7 cells after various treatments (n = 6).

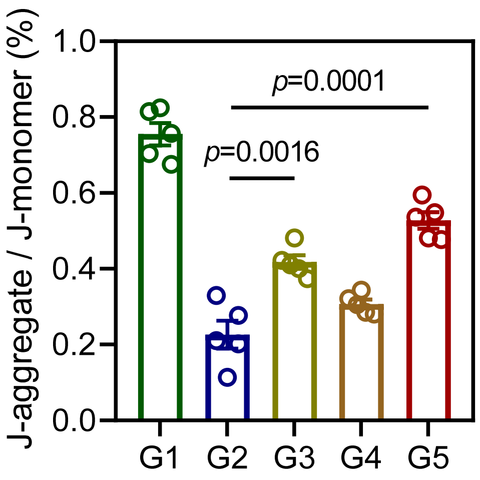

Figure S24. Corresponding quantitative analysis of the mitochondrial membrane potential (MMP) in MC3T3-E1 cells after different treatments (n = 5). G1: control group, G2: H_2O_2 group, G3: H_2O_2+Ce^{3+} group, G4: H_2O_2+Aln group, G5: H_2O_2+Ce-Aln gel group. **P* < 0.05, ***P* < 0.01, ****P* < 0.001, and *****P* < 0.0001, as determined by Student's t-test; ns, not significant.

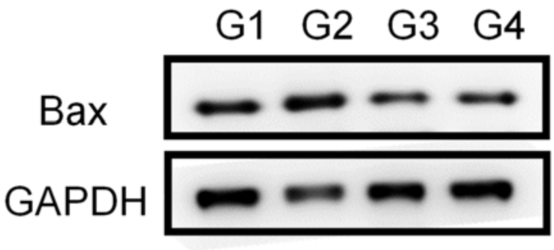

Figure S25. The expression of the apoptosis-related protein Bcl2-associated X (Bax) in MC3T3-E1 cells after different treatments was determined by Western blotting (WB). G1: control group, G2: H_2O_2 group, G3: H_2O_2+Ce^{3+} group, G4: H_2O_2+Ce-Aln gel group.

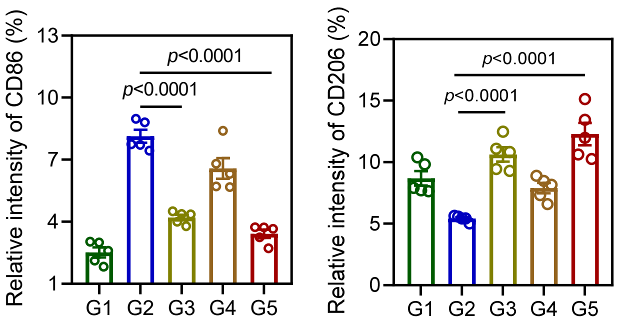

Figure S26. The expression of different inflammatory factors in macrophages after different treatments by an enzyme-linked immunosorbent assay (ELISA) kit (n = 5). G1: control group, G2: LPS group, G3: LPS+Ce^{3+} group, G4: LPS+Aln group, G5: LPS+Ce-Aln gel group. *$P < 0.05$, **$P < 0.01$, ***$P < 0.001$, and ****$P < 0.0001$, as determined by Student's t-test; ns, not significant.

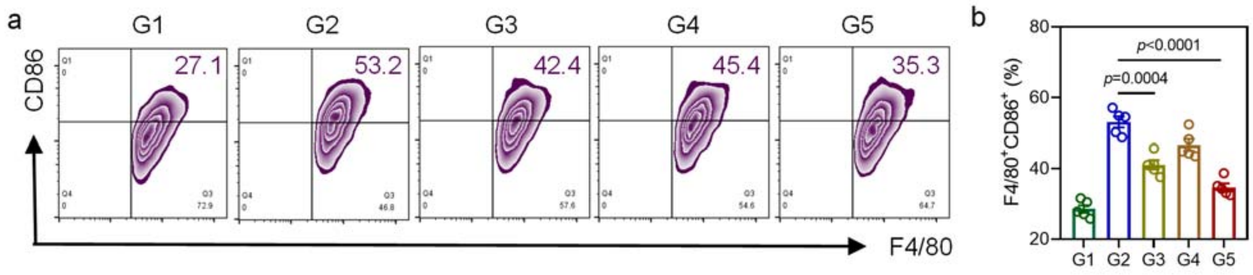

Figure S27. Evaluation of the ability of the Ce-Aln gel to regulate the M1 phenotype on RAW264.7 cells. (a) FACS results of M1 phenotype macrophages after different treatments. (b) Quantitative analysis of the FACs results of CD86 levels in a (n = 5). G1: control group, G2: LPS group, G3: LPS+Ce^{3+} group, G4: LPS+Aln group, G5: LPS+Ce-Aln gel group. *$P < 0.05$, **$P < 0.01$, ***$P < 0.001$, and ****$P < 0.0001$, as determined by a Student's t-test; ns, not significant.

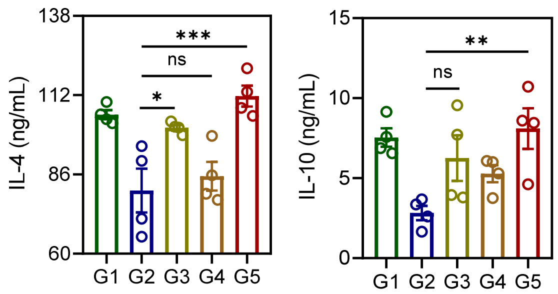

Figure S28. The expression of anti-inflammatory factors IL-4 and IL-10 in macrophages after different treatments by an enzyme-linked immunosorbent assay (ELISA) kit (n = 5). G1: control group, G2: LPS group, G3: LPS+Ce^{3+} group, G4: LPS+Aln group, G5: LPS+Ce-Aln gel group. **P* < 0.05, ***P* < 0.01, ****P* < 0.001, and *****P* < 0.0001, as determined by a Student's t-test; ns, not significant.

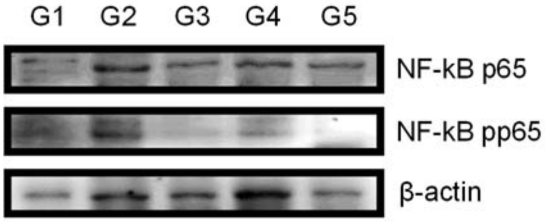

Figure S29. Expression of the NF-κB p65 and phosphorylated p65 in RAW264.7 cells after different treatments by western blotting (WB). G1: control group, G2: LPS group, G3: LPS+Ce^{3+} group, G4: LPS+Aln group, G5: LPS+Ce-Aln gel group.

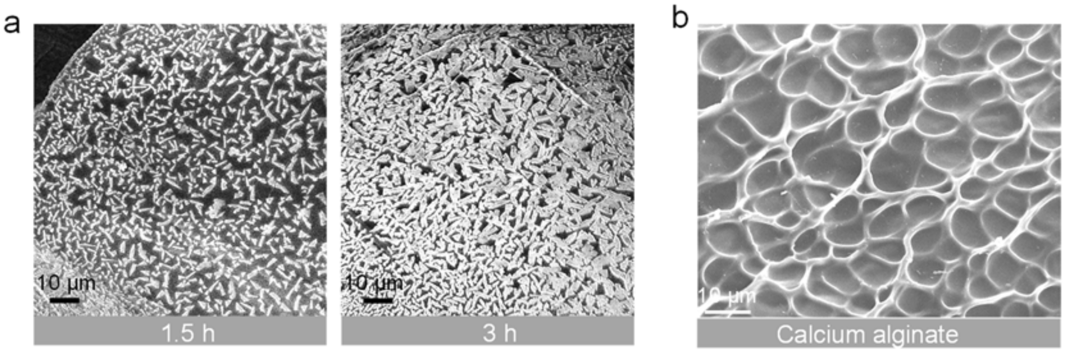

Figure S30. Detection of adsorption capacity on the surface of the Ce-Aln gel for hydroxyapatite (HAP). (a) SEM images of the Ce-Aln gel after immersion in HAP for different time. (b) SEM images of calcium alginate after immersion in HAP for 6 h.

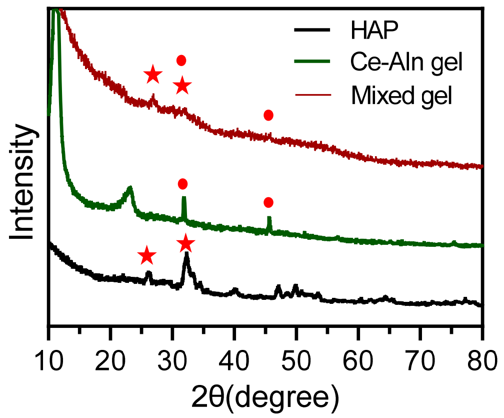

Figure S31. XRD results of HAP, Ce-Aln gel, and Ce-Aln gel immersed in HAP for 6 h (Mixed gel).

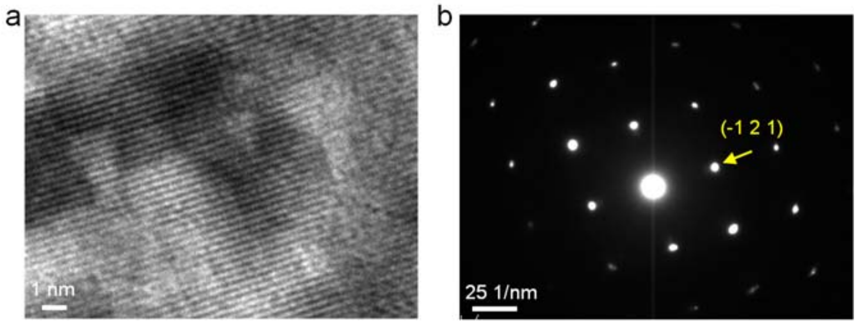

Figure S32. Characterization of the mineralized products of Ce-Aln NFs soaked in calcium ions for 3 h. (a) High-resolution TEM (HRTEM) images of the mineralized products. (b) the corresponding selected area electron diffraction (SAED) of the mineralized products.

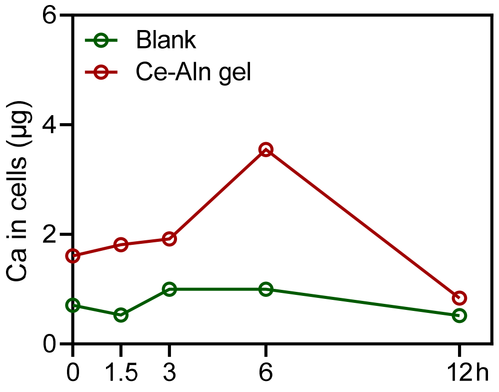

Figure S33. The concentration of intracellular calcium ions over time after different treatments (n = 3).

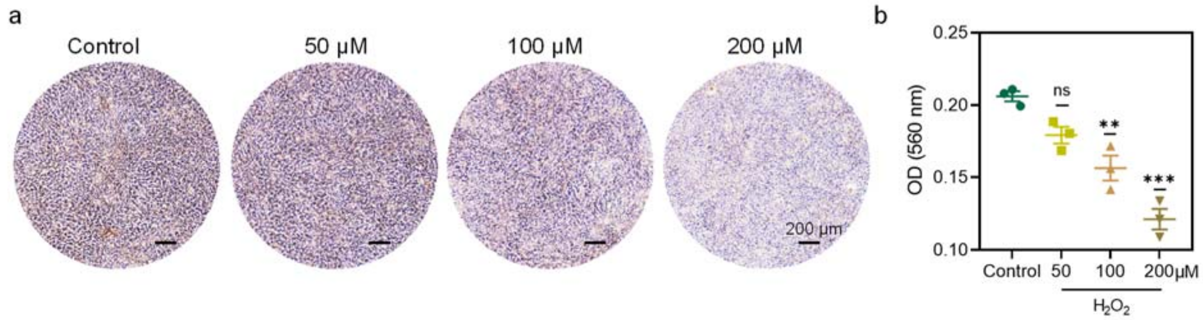

Figure S34. Detection of the osteogenic effect of H_2O_2 on the MC3T3-E1 cells for 14 days. (a)The photograph of MC3T3-E1 after different treatments stained with alizarin red S (ARS). (b) Statistical analysis of ARS staining in a (n = 3). **P* < 0.05, ***P* < 0.01, ****P* < 0.001, and *****P* < 0.0001, as determined by a Student's t-test; ns, not significant.

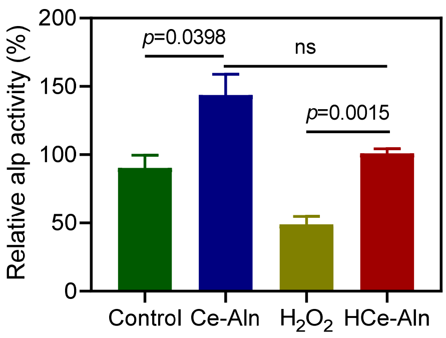

Figure S35. Statistical analysis of ALP activity in MC3T3-E1 cells after different treatments for 7 days (n = 3). **P* < 0.05, ***P* < 0.01, ****P* < 0.001, and *****P* < 0.0001, as determined by a Student's t-test; ns, not significant.

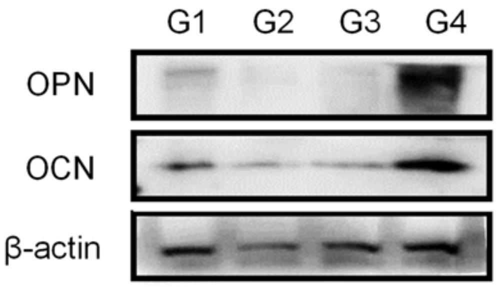

Figure S36. The expression of the osteogenic proteins osteopontin (OPN) and osteocalcin (OCN) in MC3T3-E1 cells after different treatments was determined by WB. G1: control group, G2: H_2O_2 group, G3: H_2O_2+Ce^{3+} group, G4: H_2O_2+Ce-Aln gel group.

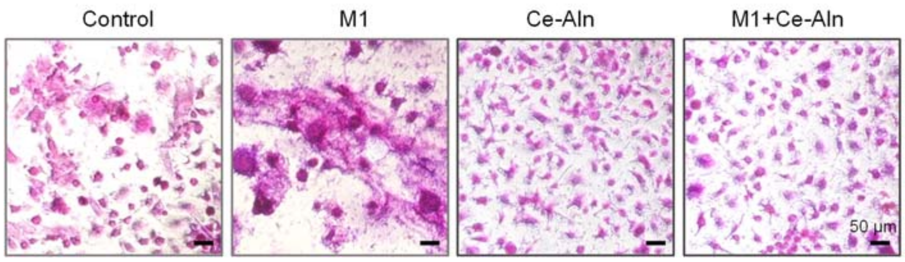

Figure S37. TRAP staining of osteoclasts induced by mouse bone marrow mononuclear cells (BMMs) after different treatments. Scale bar: 50 μm.

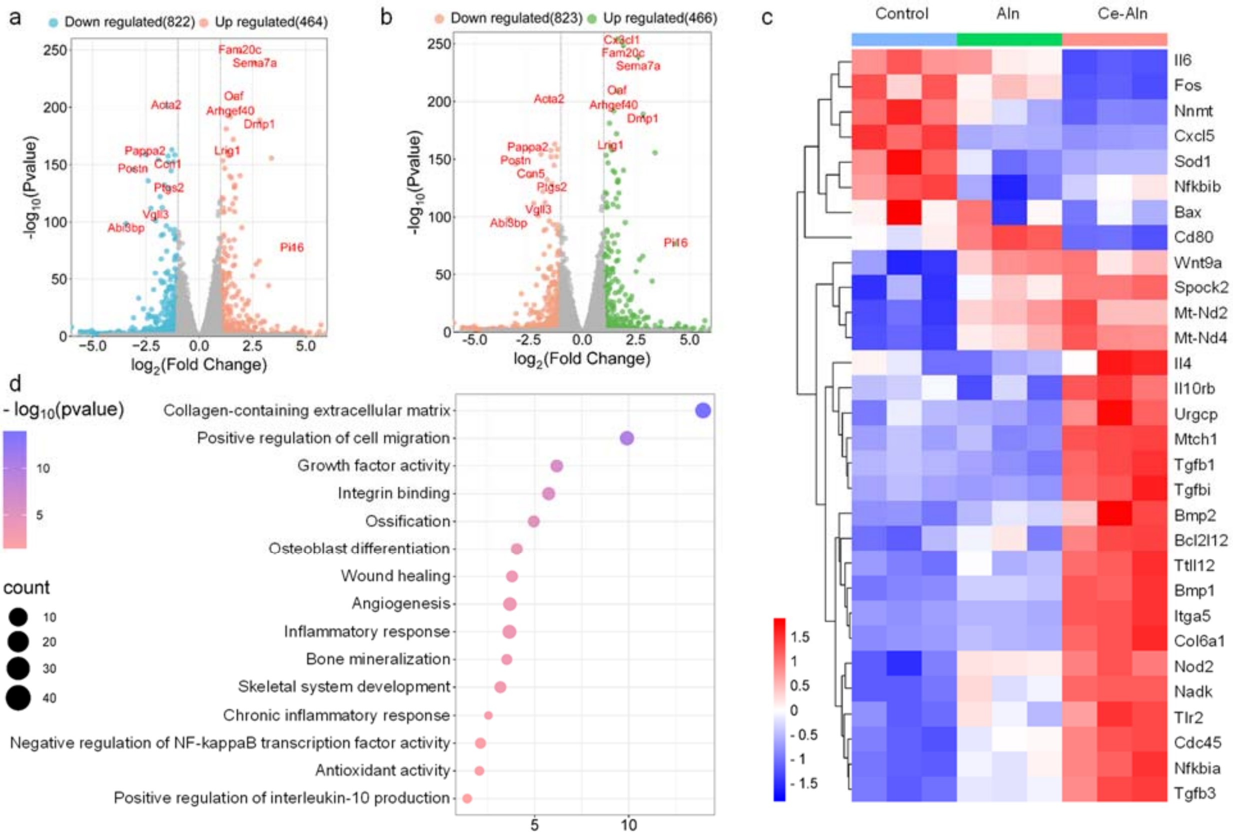

Figure S38. RNA-seq analysis of genes regulated by the Ce-Aln gel. (a) Volcano plot showing differentially expressed genes (Con vs CA). (b) Volcano plot showing differentially expressed genes between Aln and Ce-Aln gel (CA). (c) Clustering heatmap of the RNA-seq analysis results for various treatments. (Aln vs CA). (d) GO analysis of functional annotations by differentially expressed genes (Aln vs CA).

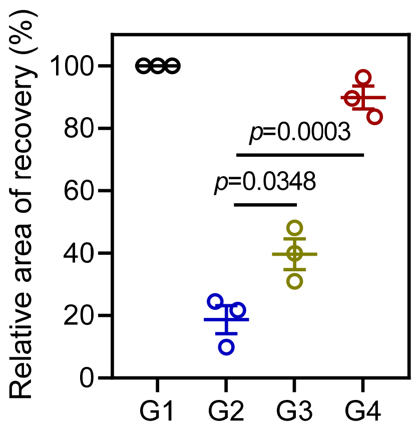

Figure S39. Statistical analysis of the relative recovery area of the defect site from the micro CT (n = 3). G1: normal group, G2: OVX group, G3: OVX+Aln group, and G4: OVX+Ce-Aln group. **P* < 0.05, ***P* < 0.01, ****P* < 0.001, and *****P* < 0.0001, as determined by a Student's t-test; ns, not significant.

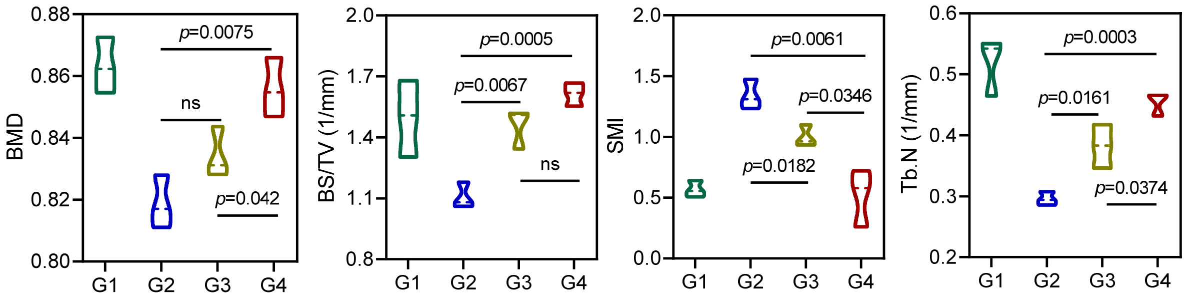

Figure S40. Statistical analysis of the relevant bone parameters of the 3D model of the ROI in osteoporotic bone defect (n = 3). G1: normal group, G2: OVX group, G3: OVX+Aln group, and G4: OVX+Ce-Aln group. **P* < 0.05, ***P* < 0.01, ****P* < 0.001, and *****P* < 0.0001, as determined by a Student's t-test; ns, not significant.

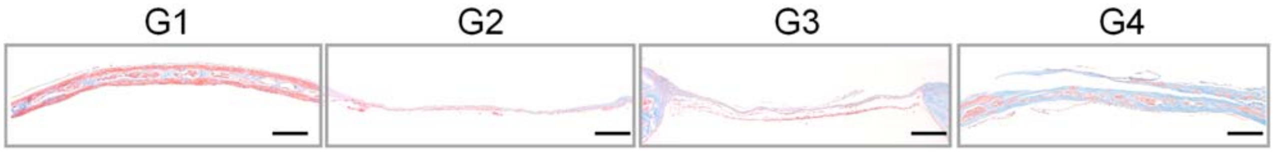

Figure S41. Masson tissue sections of the skulls from various groups. Scale bar: 300 μm. G1: normal group, G2: OVX group, G3: OVX+Aln group, and G4: OVX+Ce-Aln group.

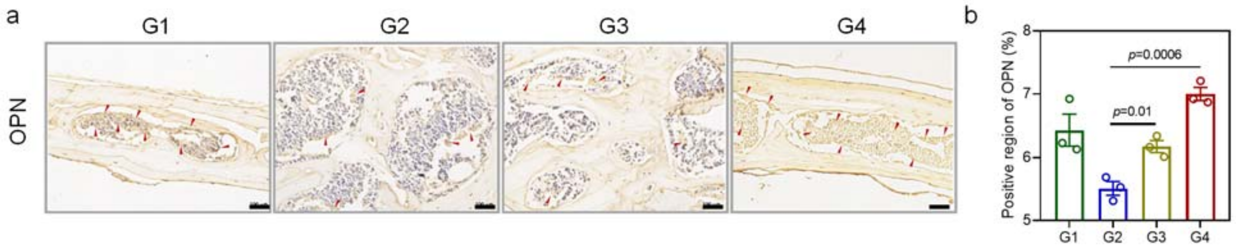

Figure S42. The expression of osteopontin (OPN) in skulls of the various groups. (a) Immunohistochemical images of OPN in the skulls of the various groups. The red arrows indicate the positive region. Scale bar: 100 μm. (b) Statistical analysis of positive region for OPN in a (n = 3). G1: normal group, G2: OVX group, G3: OVX+Aln group, and G4: OVX+Ce-Aln group. *P < 0.05, **P < 0.01, ***P < 0.001, and ****P < 0.0001, as determined by a Student's t-test; ns, not significant.

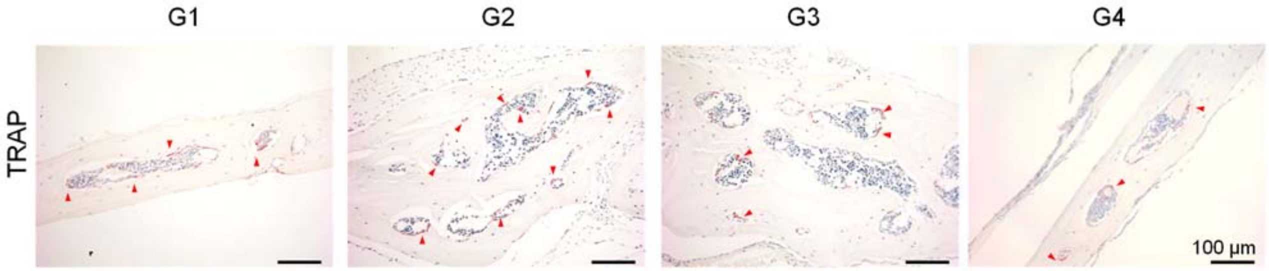

Figure S43. TRAP staining tissue section of the skull from various groups. Scale bar: 100 μm. G1: normal group, G2: OVX group, G3: OVX+Aln group, and G4: OVX+Ce-Aln group.

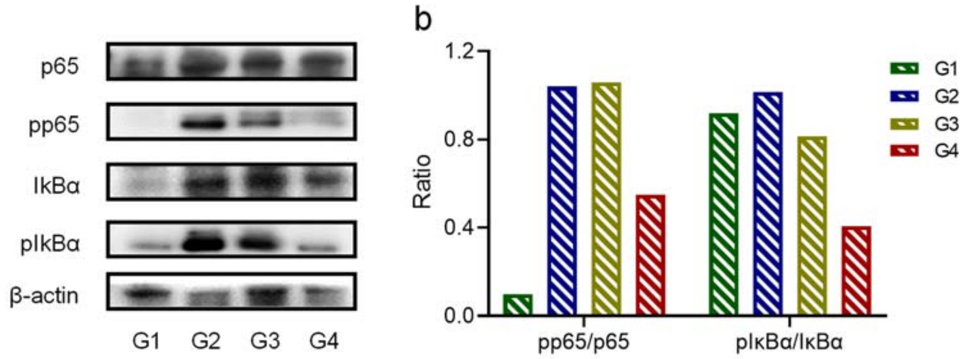

Figure S44. WB analysis of the NF-κB pathway in skull defect site tissue from the various groups at week 1. (a) The expression of the related proteins of NF-κB pathway from the various groups at week 1. (b) Quantitative analysis of NF-κB pathway-related proteins expression in a. G1: normal group, G2: OVX group, G3: OVX+Aln group, and G4: OVX+Ce-Aln group.

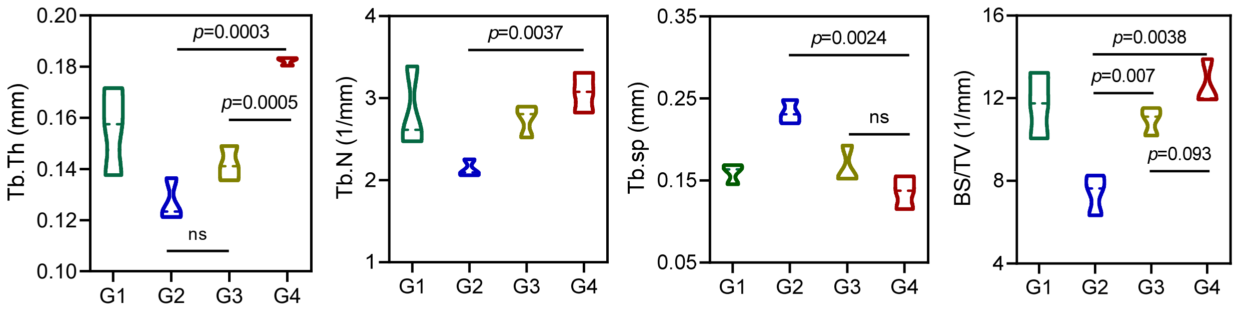

Figure S45. Statistical analysis of the relevant bone parameters of the 3D model of ROI in the micro-CT of the femur (n = 3). G1: normal group, G2: OVX group, G3: OVX+Aln group, and G4: OVX+Ce-Aln group. *$P < 0.05$, **$P < 0.01$, ***$P < 0.001$, and ****$P < 0.0001$, determined by a Student's t-test; ns, not significant.

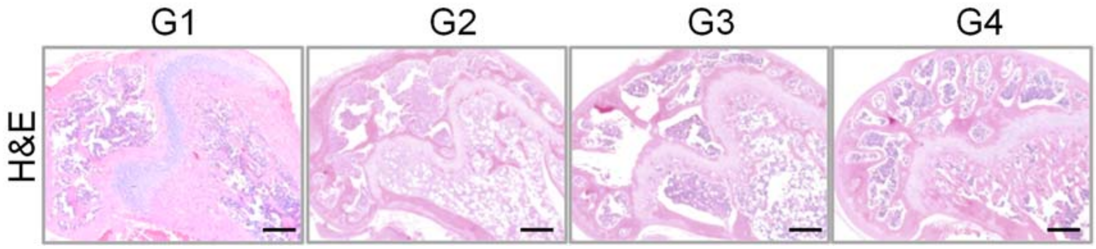

Figure S46. Hematoxylin-eosin (H&E) staining of tissue sections of the femurs from various groups. Scale bar: 300 μm. G1: normal group, G2: OVX group, G3: OVX+Aln group, and G4: OVX+Ce-Aln group.

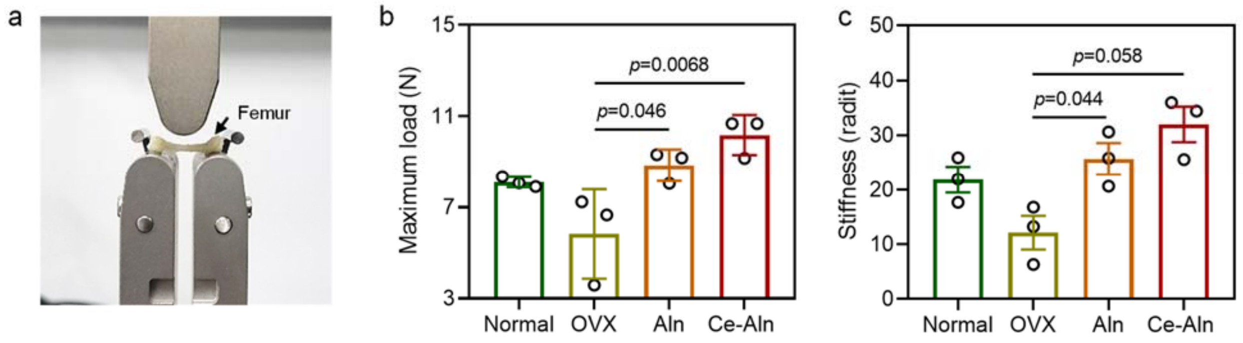

Figure S47. Biomechanical test of mouse femurs. (a) Biomechanical test diagram of mouse femurs. (b) Statistical analysis of the maximum load and (c) stiffness of mouse femurs from different groups (n = 3). *$P < 0.05$, **$P < 0.01$, ***$P < 0.001$, and ****$P < 0.0001$, as determined by a Student's t-test; ns, not significant.

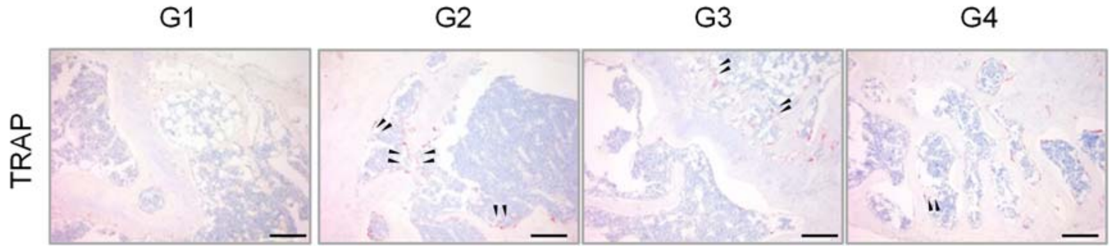

Figure S48. TRAP staining of tissue sections of the femurs from various groups. Scale bar: 100 μm. G1: normal group, G2: OVX group, G3: OVX+Aln group, and G4: OVX+Ce-Aln group.

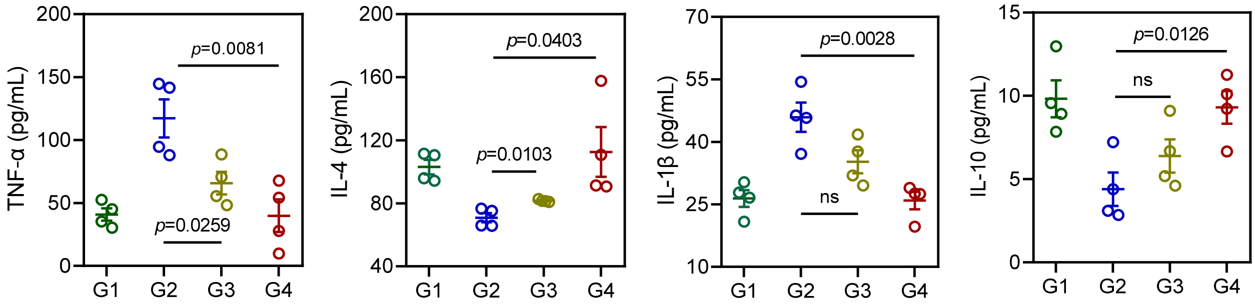

Figure S49. Evaluation of different inflammatory factors in serum from various mice (n = 4). G1: normal group, G2: OVX group, G3: OVX+Aln group, and G4: OVX+Ce-Aln group. *$P < 0.05$, **$P < 0.01$, ***$P < 0.001$, and ****$P < 0.0001$, determined by a Student's t-test; ns, not significant.

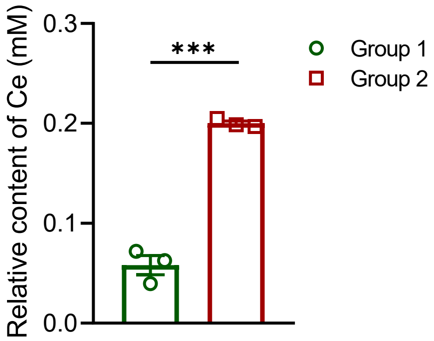

Figure S50. Ce ion concentration in plasma on day 7 after various treatments. Group 1: untreated OVX mice; Group 2: Ce-Aln gel-implanted OVX mice. *$P < 0.05$, **$P < 0.01$, ***$P < 0.001$, and ****$P < 0.0001$, as determined by a Student's t-test; ns, not significant.
